# Modeling Inter-trial Variability of Saccade Trajectories: Effects of Lesions of the Oculomotor Part of the Fastigial Nucleus

**DOI:** 10.1371/journal.pcbi.1004866

**Published:** 2016-06-28

**Authors:** Thomas Eggert, Farrel R. Robinson, Andreas Straube

**Affiliations:** 1 Department of Neurology, Ludwig-Maximilians University, Munich, Germany; 2 Department of Biological Structure, University of Washington, Seattle, Washington, United States of America; Johns Hopkins University, UNITED STATES

## Abstract

This study investigates the inter-trial variability of saccade trajectories observed in five rhesus macaques (Macaca mulatta). For each time point during a saccade, the inter-trial variance of eye position and its covariance with eye end position were evaluated. Data were modeled by a superposition of three noise components due to 1) planning noise, 2) signal-dependent motor noise, and 3) signal-dependent premotor noise entering within an internal feedback loop. Both planning noise and signal-dependent motor noise (together called accumulating noise) predict a simple S-shaped variance increase during saccades, which was not sufficient to explain the data. Adding noise within an internal feedback loop enabled the model to mimic variance/covariance structure in each monkey, and to estimate the noise amplitudes and the feedback gain. Feedback noise had little effect on end point noise, which was dominated by accumulating noise. This analysis was further extended to saccades executed during inactivation of the caudal fastigial nucleus (cFN) on one side of the cerebellum. Saccades ipsiversive to an inactivated cFN showed more end point variance than did normal saccades. During cFN inactivation, eye position during saccades was statistically more strongly coupled to eye position at saccade end. The proposed model could fit the variance/covariance structure of ipsiversive and contraversive saccades. Inactivation effects on saccade noise are explained by a decrease of the feedback gain and an increase of planning and/or signal-dependent motor noise. The decrease of the fitted feedback gain is consistent with previous studies suggesting a role for the cerebellum in an internal feedback mechanism. Increased end point variance did not result from impaired feedback but from the increase of accumulating noise. The effects of cFN inactivation on saccade noise indicate that the effects of cFN inactivation cannot be explained entirely with the cFN’s direct connections to the saccade-related premotor centers in the brainstem.

## Author Summary

In movement control, online feedback compensation of internal noise directly affects the statistics of the movement trajectory, namely the development of the variance during the movement and the correlation of the effectors position during the movement and its end position. We used here the statistics of the movement trajectory to gain inference about features of the underlying noise sources and of the actual feedback mechanism. We developed a method to analytically determine the statistics of movement trajectories resulting from noise entered inside and outside of an internal feedback loop and obtained a general model of noise in the output of feedback controlled motor systems. In detail we consider here the special case of saccade control and show that the number of free model parameters is small enough to identify the contribution of the noise components inside and outside of the loop, and the strength of the feedback. The model was fitted to the inter-trial saccade variability observed in five rhesus monkeys (Macaca mulatta). By comparing our parameter estimates for saccades under control conditions to saccades executed during inactivation of the fastigial nucleus (the main cerebellar output to the brainstem saccade generator) we quantify the role of the cerebellum in feedback motor control.

## Introduction

Biologically controlled movement may be seen as output of a dynamic system driven by non-deterministic inputs. Repeated natural movements exhibit a considerable amount of inter-trial variability even if external noise related to the stimulus, the task, and perturbing forces is minimized. This trial to trial variability indicates that movement control is subject to internally generated noise. It is important to investigate the origin of this noise and the dynamics of the system it is passed through. The primary data source for empirical research on this topic is the time course of the output variance and the covariance of the output between different time points during the movement. Such data contain relevant information since the variance/covariance structure of the output of a dynamic system driven by a random input signal is a function of the system dynamics and of the power density of the input signal. For linear systems, efficient methods to compute the variance/covariance structure of the output for a given input noise do exist. However, a number of difficulties interfere with the interpretation of inter-trial variance of natural movements. First, the input noise which is generated within the system is normally not directly observable. Second, the dynamic relationship between a noise source and the observed output is not known in detail. Third, the observed variance may result from multiple noise sources such as sensory noise, cellular noise, and peripheral motor noise [1]. The decomposition of the observed variance into its different components may not be possible without ambiguity.

In pure horizontal saccadic eye movements these problems are less pronounced. A model of peripheral motor noise for saccades already exists [2, 3] which approximates motor noise entering at the level of the oculomotor neurons (ONs) by additive white Gaussian noise with a standard deviation that scales linearly with firing rate. This model very successfully explains the saccade main sequence, i.e., the relation between saccade amplitude, peak velocity, and duration [4–6], as a consequence of minimizing variance of eye position during a post-movement period in the presence of signal-dependent motor noise. The internal movement commands in the noise model of Harris and Wolpert [2] are based on well-established dynamic properties of the oculomotor plant describing the dependence of eye position during saccades on the activity of the ON [7, 8]. The model of Harris and Wolpert [2] predicts both the time course of eye position variance and also the covariance of eye position during any two time points during the saccade. It provides a statistical model of inter-trial variability of the saccade trajectory induced by signal-dependent noise in the ONs and solves major parts of the above-mentioned difficulties associated with the modeling of motor noise.

It is important to underline that the model of Harris and Wolpert [2] was not designed to provide a statistical model of experimentally observed noise of the saccade trajectory. Its purpose was to identify the best motor plan (represented by the time course of ON activity) for minimizing end point variance. Therefore, this model explicitly excludes all noise sources that may enter the saccadic system upstream from the ONs.

The current study models inter-trial variance of saccade trajectories and can therefore not avoid the problem that the observed variance may result from multiple noise sources. Ideally, a model would allow us to decompose the noise into its different components. To that end we will start with the model of Harris and Wolpert [2] and will extend it with two different noise components that enter upstream from the ON. These extensions are motivated as follows.

First, it is known that burst-like activity related to horizontal saccades occurs not only in the ONs, but also in a number of different premotor burst neurons belonging to the so-called brainstem pulse generator [9]. Thus, it seems plausible that motor noise affects not only the activity of ONs but also the activity of other premotor burst neurons (PBNs). An essential difference between the ONs and PBNs is that ON activity is propagated feedforward through the dynamics of muscle recruitment and the mechanics of the eyeball, whereas PBN activity is also subject to internal feedback [10–15]. This difference may be very important for the variability of the saccade trajectory induced by signal-dependent motor-noise added at these different levels of the motor system. Therefore, the present study investigates how signal-dependent noise added within a premotor feedback loop affects the variability of saccade trajectories in comparison to signal-dependent noise in the ONs, downstream from the feedback loop. If these two different noise sources affect the noise structure of saccade trajectories differently, then a successful noise model could provide new insight into the relative contributions of the different noise sources and it could also provide evidence for an internal feedback loop and a quantitative estimate of its gain.

The second motivation for extending the model of saccadic motor noise of Harris and Wolpert [2] is that, in addition to motor noise, variability of the neural signals driving the brainstem saccade generator (e.g. planning noise) also affects saccades. Noise entering upstream from the motor system would cause variability of saccade trajectories; even in the absence of motor-noise (i.e. noise entering directly at the levels of saccade-related burst activity). Therefore, theoretical models of saccadic motor noise such as the model of Harris and Wolpert [2] cannot characterize the variability of experimentally observed saccades without accounting for non-motor noise affecting sensory processing, target selection, and cortical representations of the initial motor error. In the following we will summarize all noise sources that contribute to the variability of the planned saccade amplitude under the term “planning noise”.

The question of how the variability of saccades can be modeled by an explicit decomposition into different noise components was previously addressed by a study of van Beers [16]. This previous approach is extended by the current study in two ways: In the concluding remarks of his study, van Beers [16] mentioned that the exact contribution of noise entering within a premotor feedback loop to end point variance is not known. Our attempt to distinguish between motor noise that is propagated feedforward through the motor plant and premotor feedback noise is a first approach to fill this gap. The second new aspect is that the current study will model the dynamic evolution of the variance of eye position during the saccade, whereas the study of van Beers [16] is focused on modeling of end point variance and the correlation between saccade parameters (amplitude, duration, peak velocity).

The current study also compares the variability of saccade trajectories under control conditions to the variability of saccades executed during inactivation of the caudal fastigial nucleus (cFN) on one side of the cerebellum. The cFN is one of the major cerebellar structures involved in the control of visually guided saccades [17]. This comparison is of particular interest since the most prominent impairment of motor control in cerebellar lesions (not only in the control of saccades but also in the control of posture and limb movements) is the loss of precision and accuracy. According to theories of cerebellar function [18–20], these deficits are related to the role of the cerebellum in shaping feedforward motor commands and in predicting the sensory consequences of the movement. These predictions, in comparison with actual sensory afferents, can be used for driving online feedback and adaptive modifications of the system. For saccades which are not affected by visual online feedback [21–23], a number of studies [24–28] suggest that the cerebellum is involved in the above-mentioned internal feedback. However, the anatomical substrates of this internal feedback loop are not known [29]. A noise model that would allow to estimate the gain of the internal feedback in both control saccades and saccades during cFN inactivation could reveal new details about the contribution of the cerebellum to internal feedback.

## Results

### Inter-trial variability of saccades under control conditions

**Fig 1:**
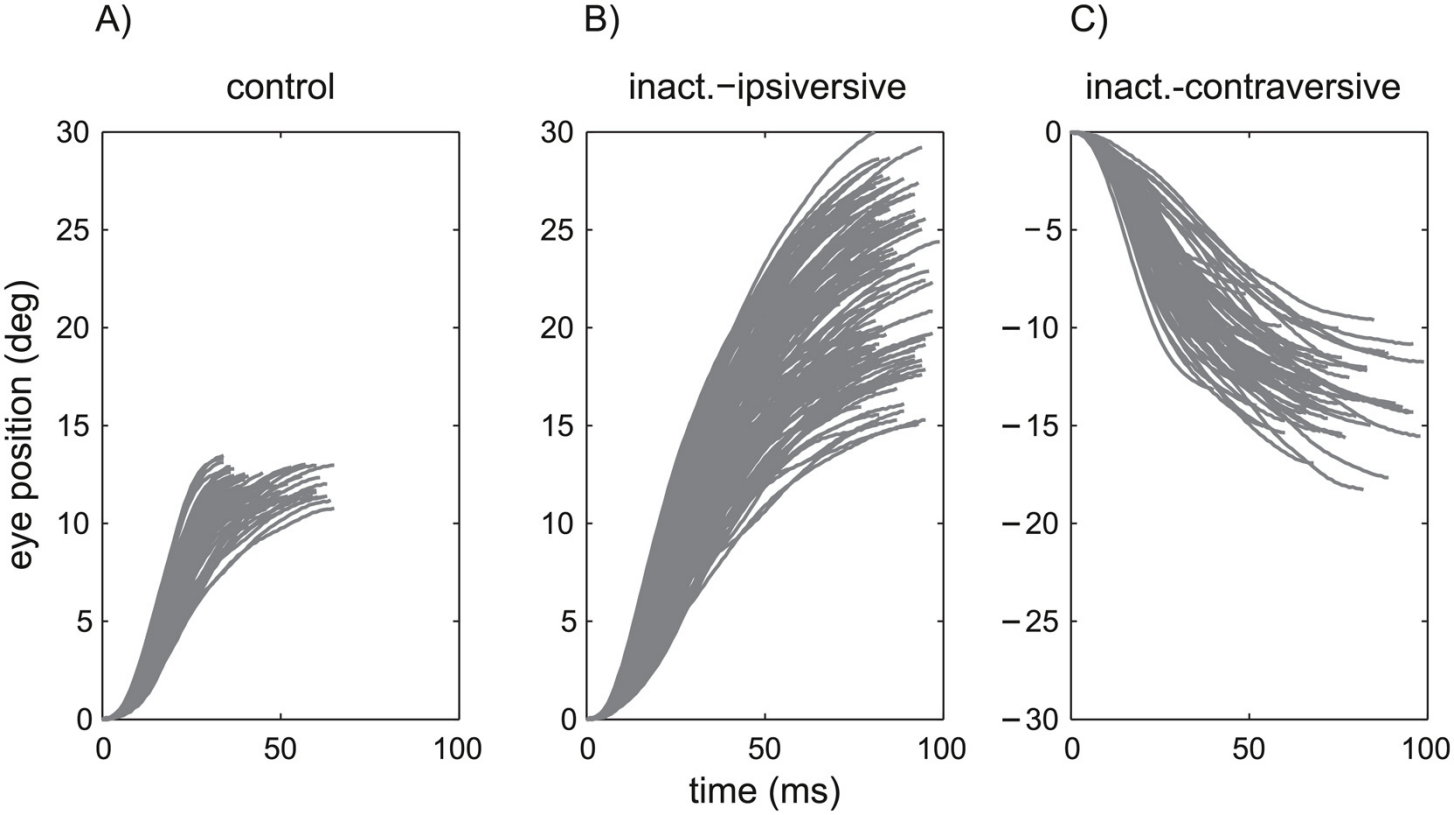
Saccades under control conditions. Eye position traces of monkey 4 during saccades under control conditions (A), and during cFN inactivation for saccades ipsiversive (B) and contraversive (C) to the side of the inactivated cFN.

Fig 1A shows the saccade trajectories of one monkey. These saccades, made under control conditions, were selected by their motor error which was restricted to 10±2.5 deg.

#### Statistics of duration and amplitude

Averaged across all monkeys, saccade amplitude was 10.1±0.2 (mean ± SD) deg and saccade duration was 40.0±2.5 ms. The inter-trial variance of the saccade duration was 34±32 ms^2^ and the inter-trial variance of the saccade amplitude was 1.6±1.2 deg^2^ (Table 1A, columns *var*_*TDur*_, *var*_*Amp*_). The correlation coefficient between duration and amplitude was only 0.17±0.19 (Table 1A, column *CC*_*TDur*,*Amp*_) and did not (except in one monkey) significantly differ from zero. This shows that the inter-trial variability of amplitude and duration was not sufficiently explained by planning noise alone. If it were (i.e. if the motor noise was negligibly small compared to planning noise), then the relationship between amplitude and duration would be determined by the main sequence. Each small deviation (Δ*Amp*) of a saccade amplitude from its mean would be roughly proportional to the corresponding deviation (*ΔTDur*) of the saccade duration from its mean: Δ*Amp = α∙ΔTdur*. Consequently, the variance of the saccade amplitude *var*_*Amp*_ would approach *α*^2^∙*var*_*TDur*_ and the coefficient of the correlation between saccade duration and amplitude would be close to one. The absence of a strong correlation between amplitude and duration in the observed data may be explained by control commands which entered the motor system independently from the planned feedforward command. Such planning-independent control commands can include motor noise destabilizing and/or online feedback stabilizing saccade amplitudes. In the absence of any feedback, motor noise would cause the variance of saccade amplitude to increase above the planning variance *α*^2^∙*var*_*TDur*_. But in contrast to this scenario the experimentally observed variance of the saccade amplitude (Table 1A, column *var*_*Amp*_) was about 10 times smaller than *α*^2^∙*var*_*TDur*_ suggesting that some type of internal feedback stabilized saccade amplitude despite considerable variance of the saccade duration.

**Table 1:** Inter-trial statistics of saccade duration and amplitude for each monkey.

A: Saccades during control conditions					
Monkey	*var*_*TDur*_ [ms^2^]	$\alpha \;\left[\frac{\text{deg}}{\text{ms}}\right]$	*α*^2^∙*var*_*TDur*_ [deg^2^]	*var*_*Amp*_ [deg^2^]	*CC*_*TDur*,*Amp*_
1 (✯)	23.5	0.58	7.8	1.5	0.44*
2 (♢)	8.2	0.57	2.7	0.2	0.28
3 (▽)	58.5	0.72	30.1	3.0	0.02
4 (◯)	75.8	0.75	43.0	2.5	0.12
5 (□)	3.9	0.66	1.7	0.7	0.00
Mean±SD	34.0±31.7	0.66±0.08	17.1±18.5	1.6±1.2	0.17±0.19
B: Ipsiversive saccades during cFN inactivation.					
Monkey	*var*_*TDur*_ [ms^2^]	$\alpha \;\left[\frac{\text{deg}}{\text{ms}}\right]$	*α*^2^∙*var*_*TDur*_ [deg^2^]	*var*_*Amp*_ [deg^2^]	*CC*_*TDur*,*Amp*_
1 (✯)	41.3	0.60	14.7	3.3	0.66*
2 (♢)	197.3	0.43	36.3	10.9	0.83*
3 (▽)	703.8	0.47	156.2	8.8	0.72*
4 (◯)	130.6	0.65	55.4	13.7	0.31*
5 (□)	244.5	0.64	100.0	19.9	0.77*
Mean±SD	263.5±257.7	0.56±0.10	72.5±56.4	11.3±6.1	0.66±0.20*
C: Contraversive saccades during cFN inactivation.					
Monkey	*var*_*TDur*_ [ms^2^]	$\alpha \;\left[\frac{\text{deg}}{\text{ms}}\right]$	*α*^2^∙*var*_*TDur*_ [deg^2^]	*var*_*Amp*_ [deg^2^]	*CC*_*TDur*,*Amp*_
1 (✯)	51.4	0.40	8.3	0.7	-0.54*
2 (♢)	167.4	0.52	45.0	2.7	-0.72*
3 (▽)	843.5	0.13	15.0	1.0	-0.58*
4 (◯)	229.8	0.52	61.3	4.8	-0.51*
5 (□)	267.9	0.28	20.6	1.9	-0.60*
Mean±SD	312.0±308.2	0.37±0.17	30.0±22.3	2.2±1.7	-0.59±0.08*

*var*_*TDur*_: variance of total saccade duration. *α*: slope of the dependency of total saccade amplitude on saccade duration according to the main sequence estimated for each monkey and saccade condition. *α*^2^∙*var*_*TDur*_: expected variance of the saccade amplitude if all saccades were to exactly obey the main sequence. *var*_*Amp*_: variance of the saccade amplitude. *CC*_*TDur*,*Amp*_: Pearson’s correlation coefficient between the total duration and the amplitude of the saccade. Significant correlations are marked by an asterisk. Inter-trial variances and correlation coefficients were computed across all available saccades with motor errors between 8.5 and 12.5 deg. In all cases, *var*_*Amp*_ was smaller than *α*^2^∙*var*_*TDur*_, and |*CC*_*TDur*,*Amp*_| was smaller than 1.

Using only the variances of amplitude and duration it is not possible to estimate the relative contributions of planning noise, motor noise, and internal feedback in more detail. To estimate these contributions we analyzed the variance and covariance trajectories and compared them to the predictions of our noise model.

#### Variance- and covariance trajectories

**Fig 2:**
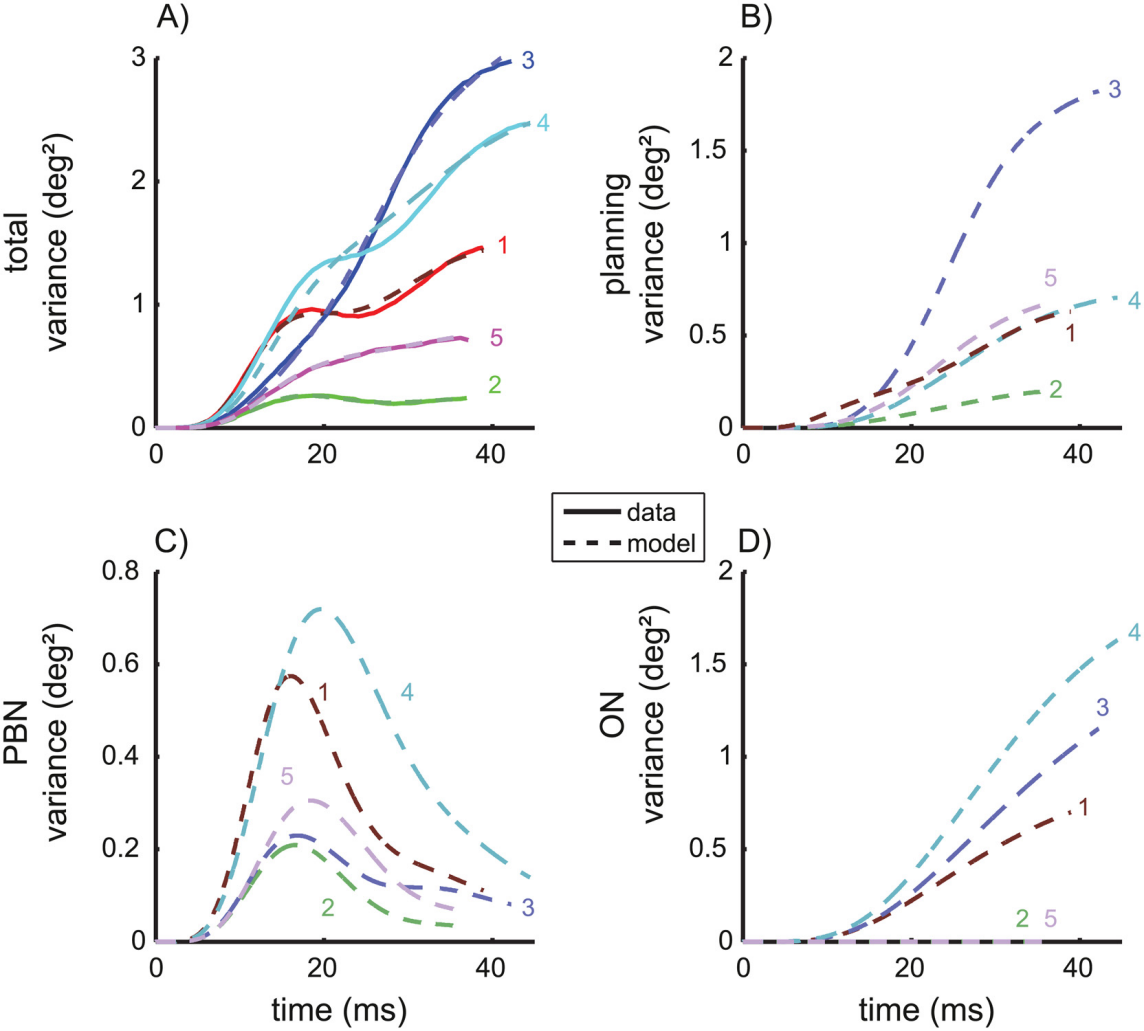
Variance trajectories under control conditions. A) Solid: measured variance of eye position during saccades with 10 deg motor error. time = 0: saccade onset. Dashed: sum of the three different components of the variance model shown in B/C/D. Variance of the eye position due to planning noise (B, Eq 10), signal-dependent noise in the premotor burst neurons (C, Eq 19), and signal-dependent noise in the oculomotor neurons (D, Eq 15).

The variance trajectories (Fig 2A, solid) show how the variance changed during the saccade. Only monkeys 3 and 5 showed a simple S-shaped increase of the variance. The trajectories of the other 3 animals showed a double S-shape. In monkeys 1 and 2 this double S-shape was strong enough to induce a local maximum in the variance occurring about 18–19 ms after saccade onset. For each monkey, we determined the four model parameters [*k*_*A*_, *k*_*ON*_, *k*_*PBN*_, *g*] that optimally fitted the measured variance/covariance trajectories. The three parameters *k*_*A*_, *k*_*ON*_, and *k*_*PBN*_ reflect the strength of the signal dependent planning noise, ON noise, and PBN noise respectively. The parameter *g* denotes the gain of the internal feedback (see Methods, Modeling noise of the saccade trajectory). This full noise model was able to explain these inter-individual differences because the time courses of the three noise components of this model differ in shape: Both planning noise (Fig 2B) and ON-motor noise (Fig 2D) showed a simple S-shape and a monotonic increase, whereas signal-dependent noise in the PBN (Fig 2C) is characterized by a bell-shaped increase-decrease pattern. The fitted superposition (Fig 2A, dashed) of the three different noise components matched the measured variance/covariance trajectories with a mean squared error (MSE) of 0.0018 deg^4^.

**Fig 3:**
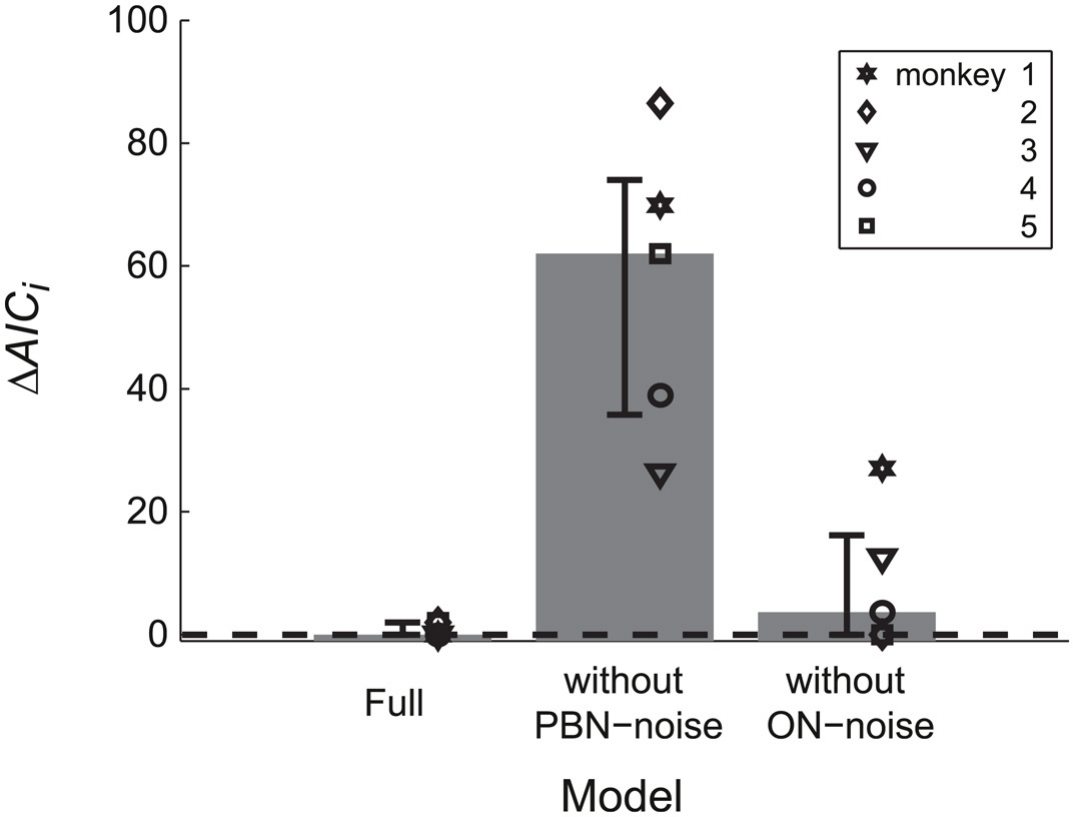
Model comparison. Model comparison between the full model and two simplified models in which the motor noise of the PBN or the ON is constrained to zero. All three models were fitted to the variance and covariance trajectories of the control saccades for each monkey (symbols). Small values of Δ*AIC*_i_ indicate little loss of explanatory value of the model with respect to the best model. Bars and whiskers indicate median and quartiles.

To test whether the different noise components of the model could be identified we fitted also two reduced models to the data in which either the ON noise or the PBN noise was constrained to zero. The MSE of the full model and those of the two reduced models differed significantly from each other (ANOVA: F(2,8) = 21.269; p<0.002). The post-hoc test showed that the MSE of the model without PBN noise (MSE = 0.0208 deg^4^), was larger (Scheffé: p<0.005) than both the MSE of the full model and of the model without ON noise (MSE = 0.0031 deg^4^). The MSE did not differ significantly between the full model and that without ON noise (Scheffé: p = 0.6). Thus, including the PBN noise improved the model, whereas omitting the ON noise did not significantly impair its ability to explain the experimentally observed noise. The same was suggested by the Akaike information criterion which showed larger values of Δ*AIC*_i_ for the model without PBN noise than for the other two models (Fig 3).

**Fig 4:**
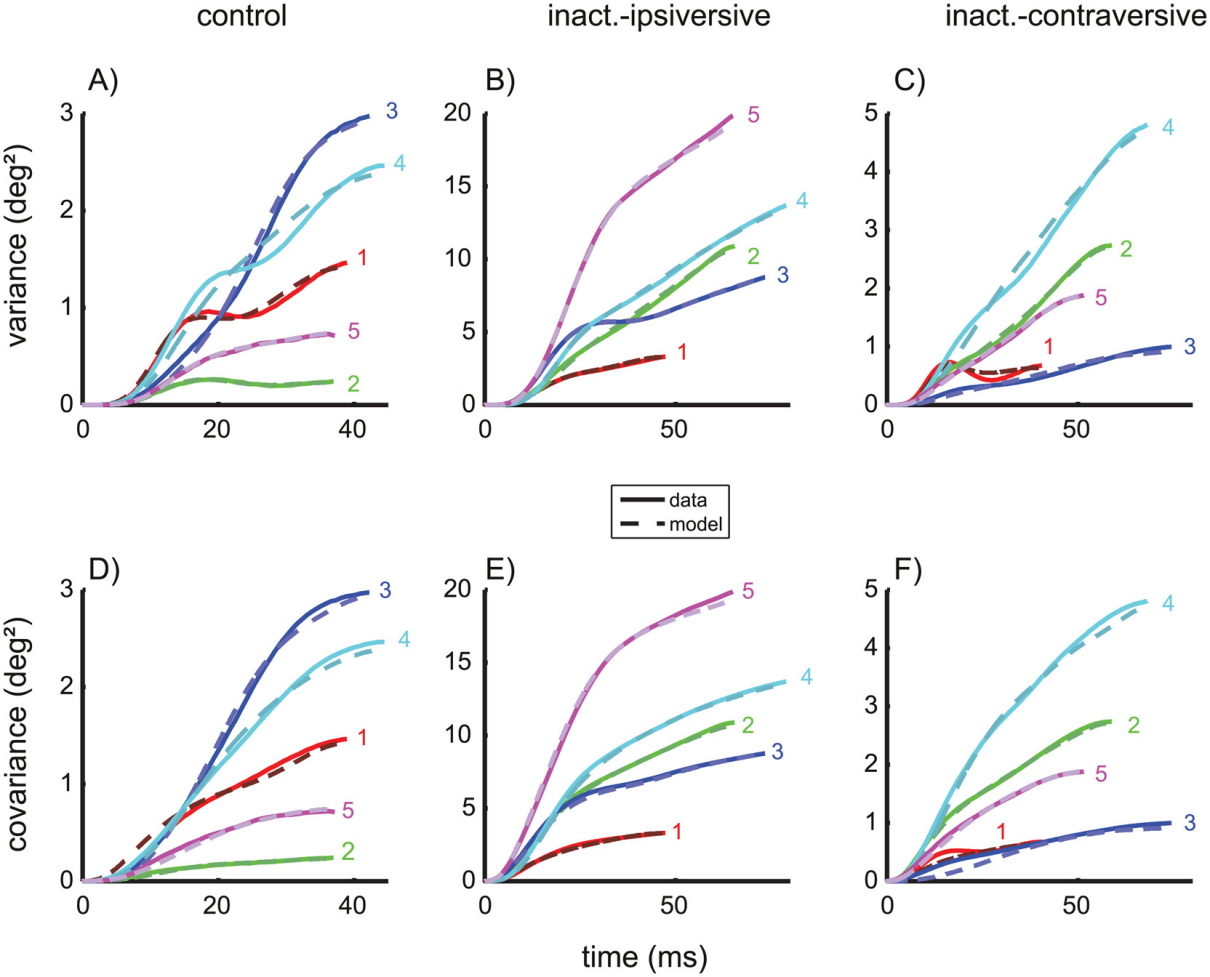
Variance- and covariance trajectories. Data (solid) and model fits (dashed) of the simplified model without ON noise (fitted parameters: [*k*_*A*_, *k*_*PBN*_, *g*]; fixed: *k*_*ON*_ = 0). The top row shows the variance trajectories for the three saccade conditions: control saccades (A), and saccades ipsiversive (B) and contraversive (C) to the side of an inactivated cFN. The bottom row (D/E/F) shows the corresponding covariance trajectories (i.e. the covariance between the eye position at the time shown on the abscissa and the eye position at the end of the saccade). Note the different scaling of the axes for each saccade condition.

The underlying cause of these results is that the shape of the variance/covariance trajectories of the planning noise and of the ON noise were similar (see Fig 2B/2D), which prevents a clear distinction between the contribution of planning noise and ON noise on the basis of the observed variance and covariance trajectories. As a consequence of this overfitting the apparent absence of ON noise in two monkeys (#2 and #5, Fig 2D) cannot be interpreted because increasing the strength of ON noise (increasing *k*_*ON*_) on the costs of planning noise (decreasing *k*_*A*_) has only minimal impact on the residual error. Since parameter estimates obtained from fitting of underdetermined models cannot be interpreted, the simplified model without signal-dependent noise in the ON (*k*_*ON*_ = 0) was used for all following estimates of the remaining parameters [*k*_*A*_, *k*_*PBN*_, *g*]. This does not mean that ON noise is assumed to be absent or irrelevant, but being unable to distinguish ON noise from planning noise, we confined ourselves to fit the saccade variability with a superposition of planning noise and PBN noise while bearing in mind that the fitted planning noise may actually represent a mixture between real planning noise and ON noise. To avoid confusion, we will therefore rename the fitted planning-noise component in the simplified model *accumulating noise* since the monotonic accumulation is the common characteristic difference of planning noise and ON noise in comparison to the bell-shaped curves of the PBN noise. The fits of the variance and covariance trajectories of control saccades achieved by the simplified model are shown in Fig 4A/4D.

**Fig 5:**
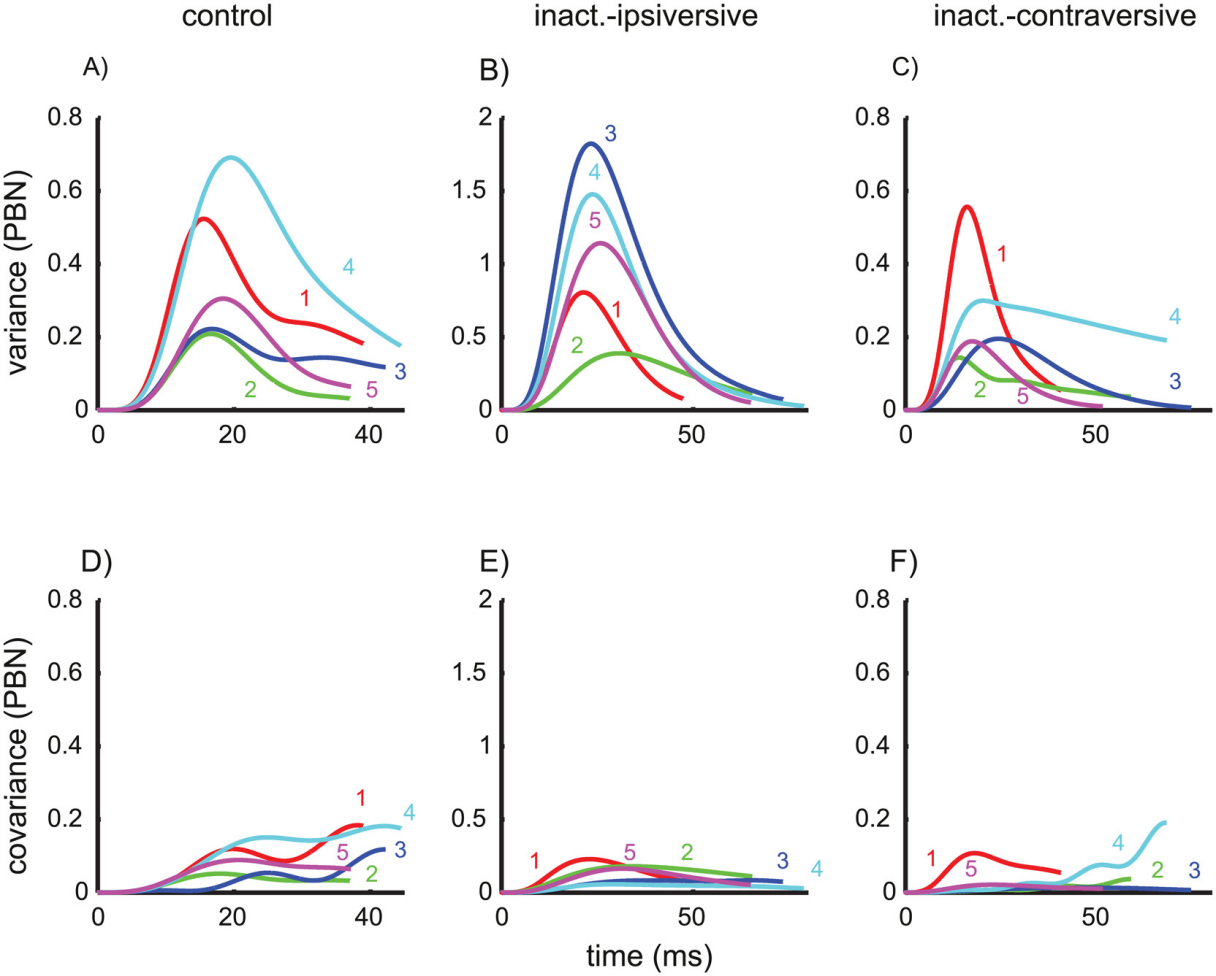
Noise component entering within the internal feedback loop. The component of the variance (A/B/C) and covariance (D/E/F) trajectories predicted by PBN noise (*r*_*PBN*_ in Fig 10) entering within the internal feedback loop. The two parameters [*k*_*PBN*_, *g*] determining this noise component were fitted together with the noise coefficient *k*_*A*_ in the simplified model without ON noise (*k*_*ON*_ fixed to zero). Fits were computed separately for each monkey and for each saccade condition. The sum of these curves and the corresponding fitted planning noise result in the dashed curves in Fig 4. Note the different scaling of the axes for each saccade condition.

The mean of the noise coefficients of the accumulating noise for the simplified model (Table 2A) was *k*_*A*_ = 0.11±0.05. Inserting the values of *k*_*A*_ (Table 2A) and the observed saccades amplitudes in Eq 12 for each monkey revealed that the variance of the saccade amplitude that the model attributes to accumulating noise (1.5±1.1 deg^2^) amounted to 94% of the total variance of the observed amplitude 1.6 deg^2^ (Table 1, *var*_*Amp*_). In the simplified model, the variability of eye position due to PBN noise (Fig 5A) showed peak variances of 0.4±0.2 deg^2^ and end point variances of only 0.11±0.07 deg^2^. This indicates that the noise component attributed to PBN noise (even though this component contributed significantly to the overall explanatory value of the model) was small compared to the component attributed to accumulating noise. The coefficients of determination were very close to one (Table 2A, R^2^), underlining the goodness of the fits.

**Table 2:** A. Parameters of the simplified noise model without ON noise (Fig 10; *k*_*ON*_ = 0).

A: Saccades during control conditions.				
Monkey	*k*_*A*_	*k*_*PBN*_	*g* [1/s]	R^2^
1 (✯)	0.102	0.032	280	0.986
2 (♢)	0.046	0.020	204	0.992
3 (▽)	0.170	0.028	317	0.997
4 (◯)	0.134	0.040	227	0.989
5 (□)	0.080	0.022	171	0.994
Mean±SD	0.106±0.048	0.028±0.008	240±59	0.992±0.004
B: Ipsiversive saccades during cFN inactivation.				
Monkey	*k*_*A*_	*k*_*PBN*_	*g* [1/s]	R^2^
1 (✯)	0.142	0.024	92	0.996
2 (♢)	0.223	0.015	35	0.998
3 (▽)	0.144	0.049	168	0.998
4 (◯)	0.183	0.035	93	0.999
5 (□)	0.184	0.023	84	0.999
Mean±SD	0.175±0.034	0.029±0.013	94±48	0.998±0.001
C: Contraversive saccades during cFN inactivation.				
Monkey	*k*_*A*_	*k*_*PBN*_	*g* [1/s]	R^2^
1 (✯)	0.094	0.034	199	0.897
2 (♢)	0.201	0.034	286	0.999
3 (▽)	0.199	0.060	110	0.940
4 (◯)	0.164	0.039	282	0.994
5 (□)	0.259	0.043	165	0.997
Mean±SD	0.183±0.060	0.042±0.011	208±76	0.965±0.045

*k*_*A*_: coefficient of variation of the planning noise Eq (12). *k*_*PBN*_: noise coefficient of signal-dependent noise in the pre motor burst neuron. g: gain of the internal feedback loop. R^2^: coefficient of determination. The feedback gain was significantly smaller for ipsiversive saccades during cFN inactivation (Table 2B) than under control conditions (Table 2A).

### Effects of cFN inactivation on saccade gain and main sequence

**Fig 6:**
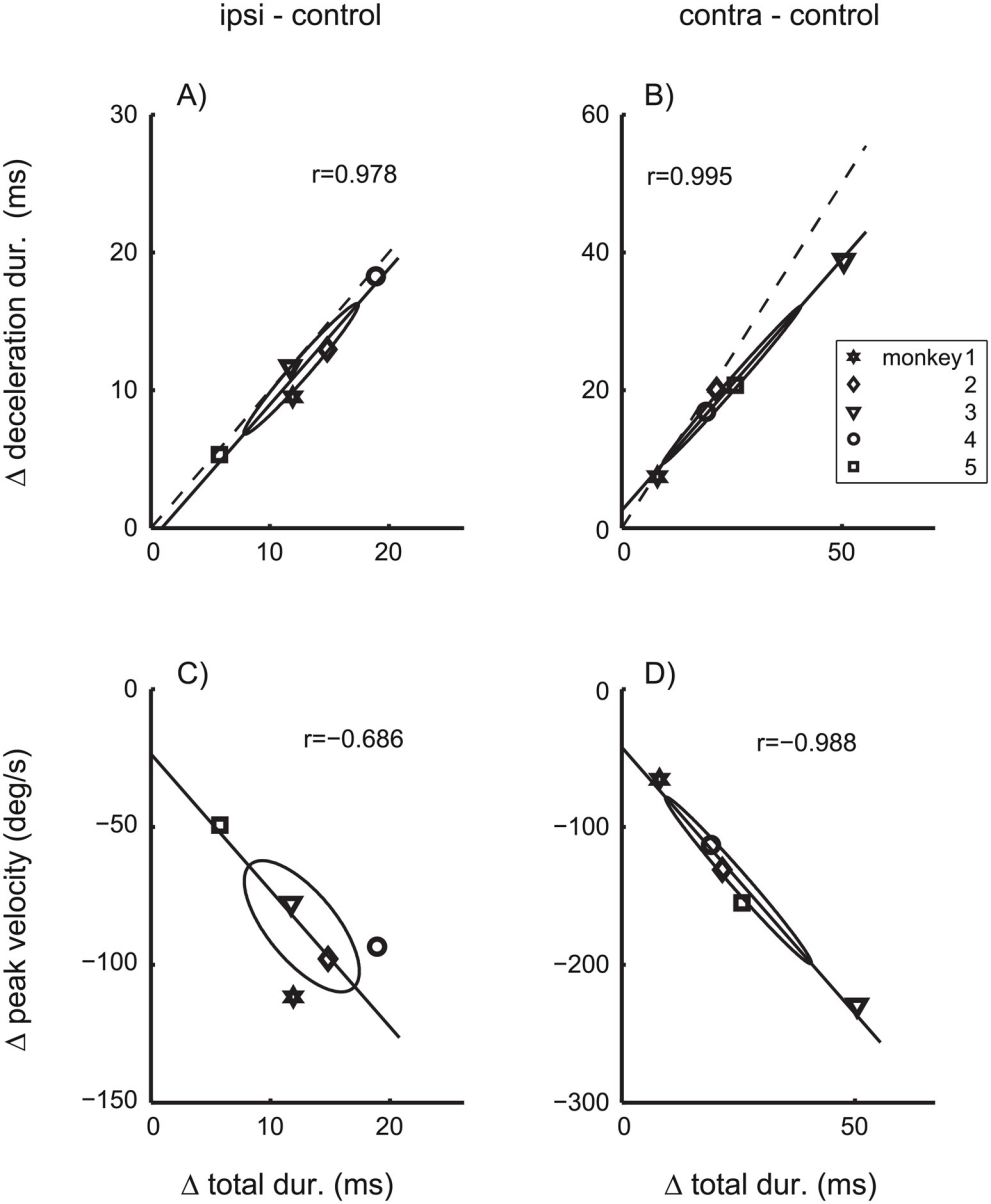
Effects of cFN inactivation on saccade dynamics. Differences of parameters of saccades with 10 deg amplitude between cFN inactivation and control condition. Inactivation effects on the duration of the deceleration phase (Δ deceleration dur.) and on the peak velocity (Δ peak velocity) are plotted versus the effect on the total saccade duration (Δ total dur.) of saccades ipsiversive (A/C) and contraversive (B/D) to the lesion side. Symbols indicate data of individual monkeys (using the same symbols as in the Tables). The covariance ellipses show large coefficients of correlation (Pearson’s r) and have their largest main axes passing close to the origins of the plots. Dashed: Lines through the origin with unity slope. cFN-inactivation effects on the different aspects of saccade dynamics were roughly proportional to each other.

As reported previously [17, 30, 31], saccades ipsiversive to the side of an inactivated cFN were hypermetric and those contraversive to it were hypometric (Table 3, ‘Amplitude’). Also the increase in the total duration of the saccade and in the duration of the deceleration phase (i.e. the time between the peak velocity and the saccade end) is fully compatible with these previous reports. However, pooling saccade duration and peak velocity across saccades to targets with the same eccentricity has the disadvantage that effects of cFN inactivation on these parameters reflect the effects on both the saccade gain and the main sequence. Therefore, to assess the effect on the main sequence independently of the effects on the saccade gain, we evaluated peak velocity, total duration, and deceleration duration for saccades with amplitudes of 10 deg (Table 3, columns 3–5). The motor errors necessary to evoke saccades with 10 deg amplitude differed across the saccade conditions (control: 9.9±0.2, ipsiversive: 5.9±1.6, contraversive: 14.3±4.2 deg), due to the saccade dysmetria induced by the inactivation of the cFN. The differences of peak velocity, total duration, and deceleration duration between Table 3B/3C and 3A reflect changes of the main sequence and the saccade velocity profile independent of changes of the saccade gain (see Methods, Data analysis, Mean saccade trajectory). Fig 6 shows the distribution of the components of effects on the velocity profile (ΔVP) across monkeys. The coefficients of correlation between these effects were all larger than 0.68, differed significantly (p<0.01) from zero for Fig 6A/6B/6D, and the major axes of all covariance ellipses pointed towards the origin. This is a critical feature for the distribution of random variables that are proportional to each other. The proportionality indices of Δ*VP* were *I*_*prop*_ = 0.68 for ipsilateral, and *I*_*prop*_ = 0.96 for contralateral inactivation effects. These results show that the three components of ΔVP were roughly proportional to each other, and that for each of the five monkeys the distortions of the velocity profile induced by cFN inactivation were dominated by a single parametric factor quantifying the strength of this distortion in each monkey.

**Table 3:** Parameters.

A: Parameters of saccades during control conditions.				
Monkey	Amplitude [deg]	Peak Velocity [deg/s]	Total Duration [ms]	Deceleration Duration [ms]
1 (✯)	10.3	468.9	36.5	19.7
2 (♢)	10.1	417.0	38.6	20.7
3 (▽)	9.9	434.2	40.9	22.9
4 (◯)	10.3	402.8	42.9	24.0
5 (□)	10.0	425.1	41.2	22.5
Mean±SD	10.1±0.2	429.6±24.8	40.0±2.5	22.0±1.7
B. Parameters of ipsiversive saccades during cFN inactivation.				
Monkey	Amplitude [deg]	Peak Velocity [deg/s]	Total Duration [ms]	Deceleration Duration [ms]
1 (✯)	12.9	357.2	48.4	29.2
2 (♢)	14.0	319.2	53.4	33.7
3 (▽)	20.8	356.4	52.7	34.6
4 (◯)	17.7	309.2	61.8	42.2
5 (□)	25.4	375.8	47.0	27.9
Mean±SD	18.1±5.1	343.6±28.1	52.6±5.8	33.5±5.7
C. Parameters of contraversive saccades during cFN inactivation.				
Monkey	Amplitude [deg]	Peak Velocity [deg/s]	Total Duration [ms]	Deceleration Duration [ms]
1 (✯)	8.4	403.4	44.6	27.1
2 (♢)	8.3	285.9	60.1	40.7
3 (▽)	5.1	204.8	91.4	61.8
4 (◯)	10.0	289.9	62.1	40.9
5 (□)	5.6	270.0	67.0	43.3
Mean±SD	7.5±2.1	290.8±71.6	65.0±16.9	42.8±12.4

Amplitudes are given for saccades with a motor error of 10 deg. All other parameters (peak velocity, saccade duration, and the duration of the deceleration phase) are specific for saccade amplitudes of 10 deg.

**Fig 7:**
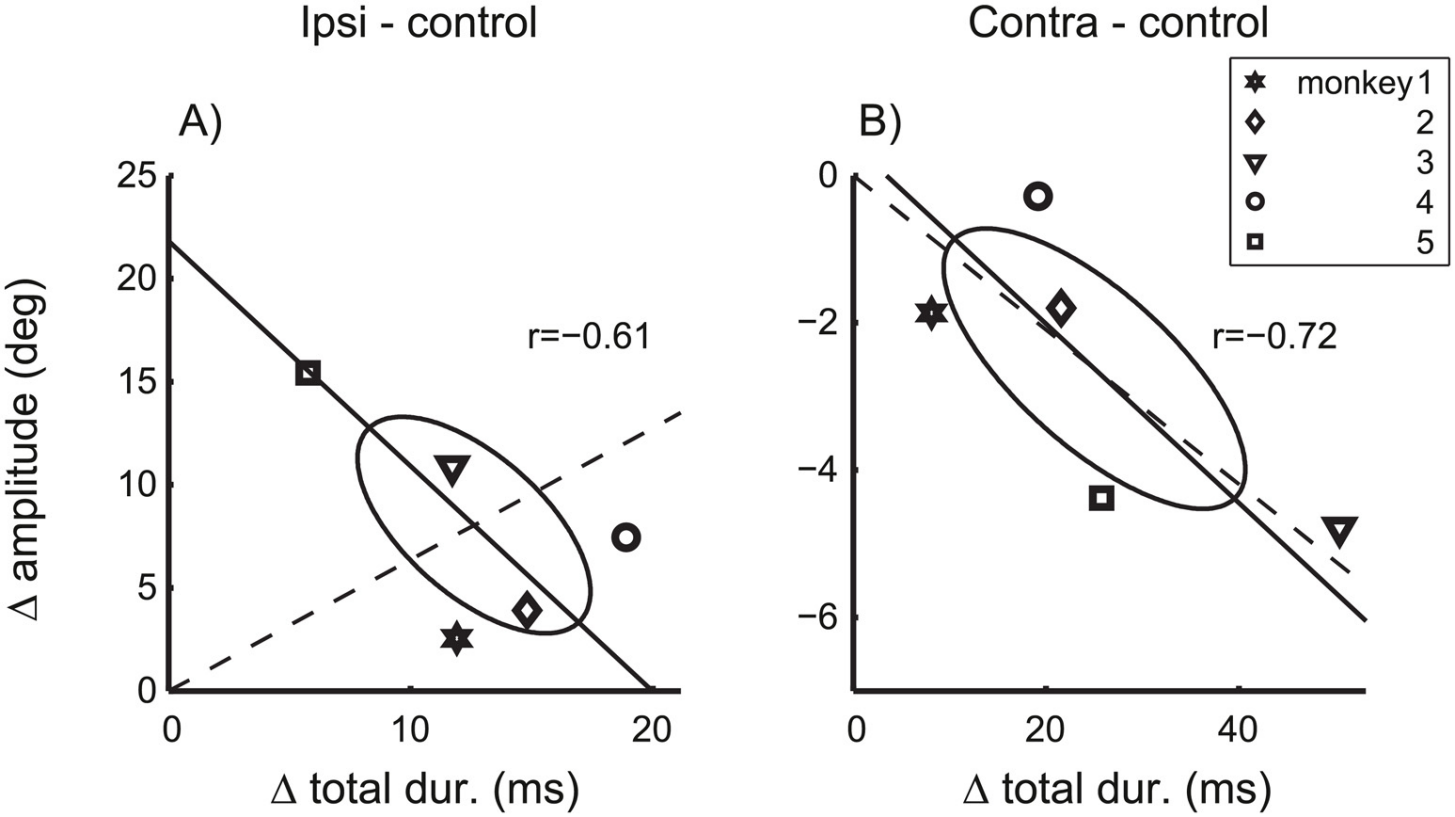
Effects of cFN inactivation on saccade gain and saccade dynamics. Δ amplitude: Amplitude differences of saccades with 10 deg motor error. Δ total dur.: Duration differences of saccades with 10 deg amplitudes. Differences were computed between saccades during cFN inactivation and control conditions and are shown separately for ipsiversive (A) and contraversive (B) saccades. Symbols denote individual monkeys. Dashed: connecting line between the origin and the mean across monkeys. The strong deviation between dashed and solid lines in A) indicates to a dissociation of effects on gain and dynamics.

If the inactivation effects on saccade amplitude were dominated by the same (monkey specific) factor, the inactivation effects on saccade amplitude (Δamplitude) should also be roughly proportional to the effects on saccade duration (Δtotal dur.). Depending on the sign of the effect on amplitude this would predict either positive (ipsilateral) or negative (contralateral) coefficients of correlations between Δtotal dur. and Δamplitude. Fig 7 shows that this expectation was not confirmed. The coefficients of correlation did not differ significantly from zero (p>0.1) for either ipsilateral (Fig 7A) or contralateral (Fig 7B) inactivation effects. For contraversive saccades, the proportionality index of the total effect vector (*I*_*prop*_ = 0.83) was only slightly smaller than that of Δ*VP* only (*I*_*prop*_ = 0.96). In contrast, for ipsiversive saccades, the proportionality index of the total effect vector (*I*_*prop*_ = 0.22) decreased to about a third of that of Δ*VP* only (*I*_*prop*_ = 0.68). Thus, the inactivation effects on the saccade amplitude were roughly proportional to the effects on the velocity profile for contraversive saccades, but not for ipsiversive saccades. For example, Fig 7A shows that monkey 5 (Symbol □) showed the strongest effect on saccade amplitude but at the same time the smallest effect on the velocity profile of 10 deg saccades. These results suggest that, for ipsiversive saccades, cFN inactivation effects on the saccade gain dissociate from those on saccade dynamics, and that both of these inactivation effects vary independently across monkeys.

### Effects of cFN inactivation on inter-trial saccade variability

#### Saccade amplitude

In the example saccades in Fig 1, the end-point variability of ipsiversive saccades during cFN inactivation (Fig 1B) was significantly larger than that of the control condition (Fig 1A). The variability of contraversive saccades (Fig 1C) was less affected by the cFN inactivation than the variability of ipsiversive saccades. The corresponding group statistics on the inter-trial variance of the saccade amplitude (Table 1A/1B/1C column *var*_*Amp*_) showed that this variance differed significantly between conditions (three levels: control, ipsiversive-inact., contraversive-inact.; repeated measures ANOVA on log(*var*_*Amp*_): F(2,8) = 9.35; p<0.01). More specifically, the variance of the amplitude of ipsiversive saccades during cFN inactivation (mean: 11.3 deg^2^) was larger (Scheffé post hoc: p<0.02) than that of control saccades (1.6 deg^2^) or contraversive saccades during cFN inactivation (2.2 deg^2^). The inactivation effect on *var*_*Amp*_ of the contraversive saccades was not significant (Scheffé: p = 0.71). During cFN inactivation, the correlation between saccade duration and amplitude (Table 1B/1C, column *CC*_*TDur*,*Amp*_) differed significantly from zero and was larger than under control conditions. This indicates that, during cFN inactivation, saccade amplitude was less stabilized against variations in saccade duration. This decreased compensation for saccade duration is consistent with reduced gain in the internal feedback (see next section).

#### Variance- and covariance trajectories

Comparing the variance and covariance trajectories of control saccades (Fig 4A/4D) and saccades during cFN inactivation (Fig 4B/4E and 4C/4F) shows that these time courses differed in their shape and not just by a scaling in time and/or amplitude. This difference indicates that the effects of cFN inactivation on the entire variance structure can provide additional information about the cFN's role in the underlying control mechanisms. Even though the effects of inactivation seem to be quite variable from monkey to monkey, the good fit of the model (dashed) to the data (solid) and the model in Fig 4 shows that appropriate adjustments of the three parameters [*k*_*A*_, *k*_*PBN*_, *g*] (Table 2) of the simplified noise model can well approximate the variance structure for all monkeys in all saccade conditions.

**Fig 8:**
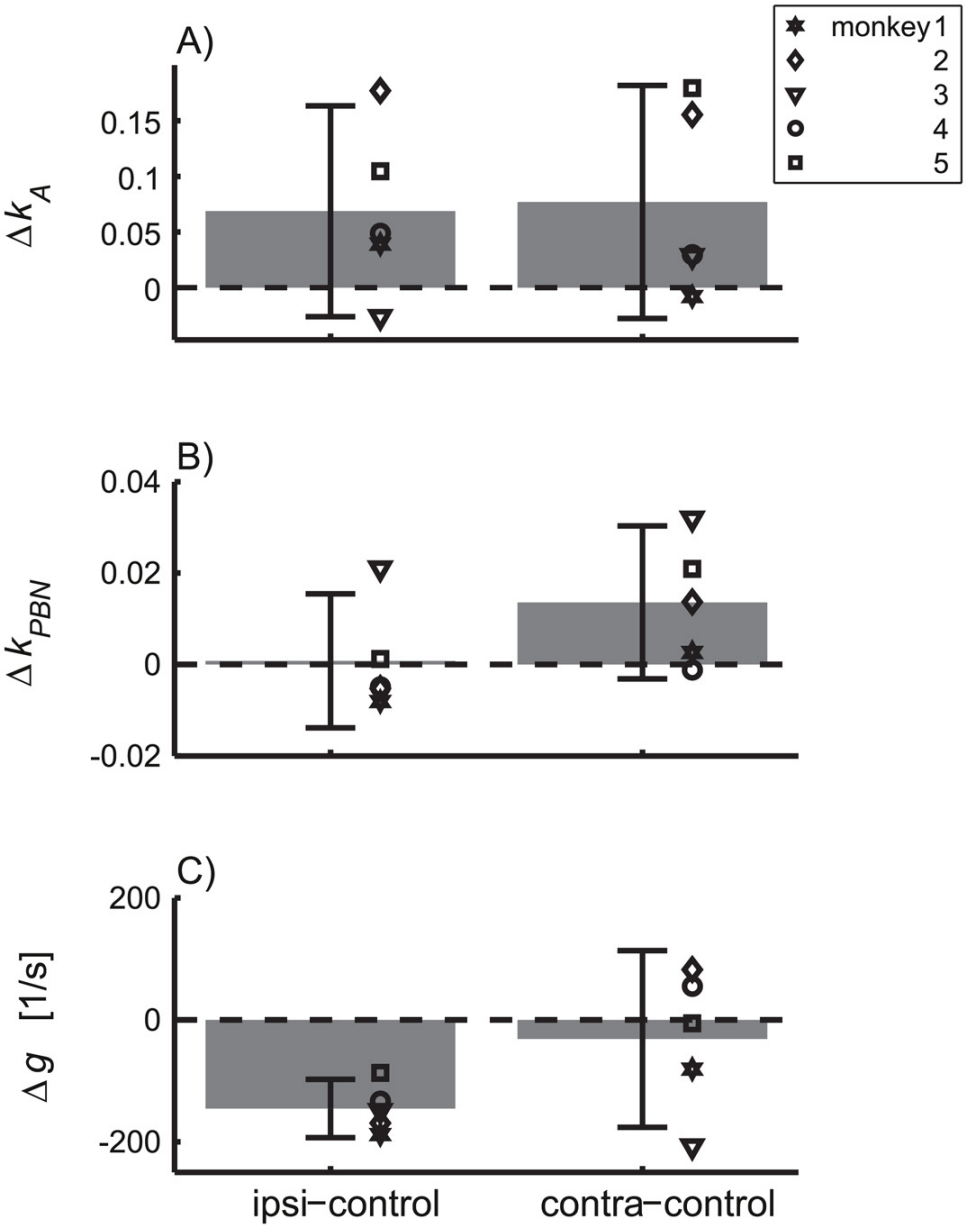
Effects of cFN inactivation on fitted model parameters. Differences of the three fitted parameters [*k*_*A*_, *k*_*PBN*_, *g*] of the noise model without ON noise between saccades during cFN inactivation (left bars: ipsiversive, right bar: contraversive) and control saccades. Bars and whiskers indicate mean ± 95% confidence interval of the mean. The gains (*g*) of the internal feedback loop (fitted to explain the observed variance and covariance trajectories) were smaller for ipsiversive saccades during cFN inactivation than for control saccades (C, left bar).

The paired differences of the fitted model parameters between saccades during cFN inactivation and control saccades (Fig 8) reveals that the feedback gain (*g*) of ipsiversive saccades during cFN inactivation was 145±39 s^-1^ smaller (T(4) = -8.43; p<0.002) than that of control saccades (Fig 8C, left bar). However, cFN inactivation did not completely prevent internal feedback in ipsiversive saccades since the feedback gain (94±48 s^-1^; Table 2B, column *g*) fitted to this saccade condition was still significantly larger than zero (T(4) = 4.43; p<0.02). None of the other effects of ipsilateral cFN inactivation on the fitted model parameter shown in Fig 8A/8B (Δ*k*_*A*_, Δ*k*_*PBN*_) differed significantly from zero or correlated significantly with the effect on the feedback gain (*ρ*(Δ*g*, Δ*k*_*A*_) = 0.08; p = 0.9; *ρ*(Δ*g*, Δ*k*_*PBN*_) = −0.61; p = 0.3).

Even though cFN inactivation effects on Δ*k*_*A*_ did not reach significance on the group level, the individual sizes of the ipsilateral Δ*k*_*A*_ ranged up to 0.18 (Fig 8A), corresponding to effects on the end point variance of up to 3.2 deg^2^ (= 0.18^2^·10^2^ deg^2^, see Eq 12). Therefore, given the high values of R^2^, the large variability of the effects on Δ*k*_*A*_ seems to reflect behavioral differences between monkeys. To analyze this hypothesis in more detail, we performed a model comparison between the simplified model (fitted parameters: [*k*_*A*_, *k*_*PBN*_, *g*], Table 2B) and an even further reduced model in which only the two parameters [*k*_*PBN*_, *g*] were fitted, and the noise coefficient *k*_*A*_ was fixed to the values obtained from the fit to the control condition (Table 2A, column *k*_*A*_). Both models were fitted to the observed ipsiversive variance/covariance trajectories during cFN inactivation. For the model with fixed *k*_*A*_, the *AIC* differences were larger than 30 in four of five monkeys (all except monkey 3). In contrast, for the model with free *k*_*A*_, the *AIC* differences were smaller than 2. This shows that for all but one animal the empirical data did not support the model with fixed *k*_*A*_. Four monkeys showed an increase of *k*_*A*_ during cFN inactivation for ipsiversive saccades. This indicates that, for ipsiversive saccades of these four monkeys, cFN inactivation induced an increase of the planning noise.

#### Correlation trajectories

**Fig 9:**
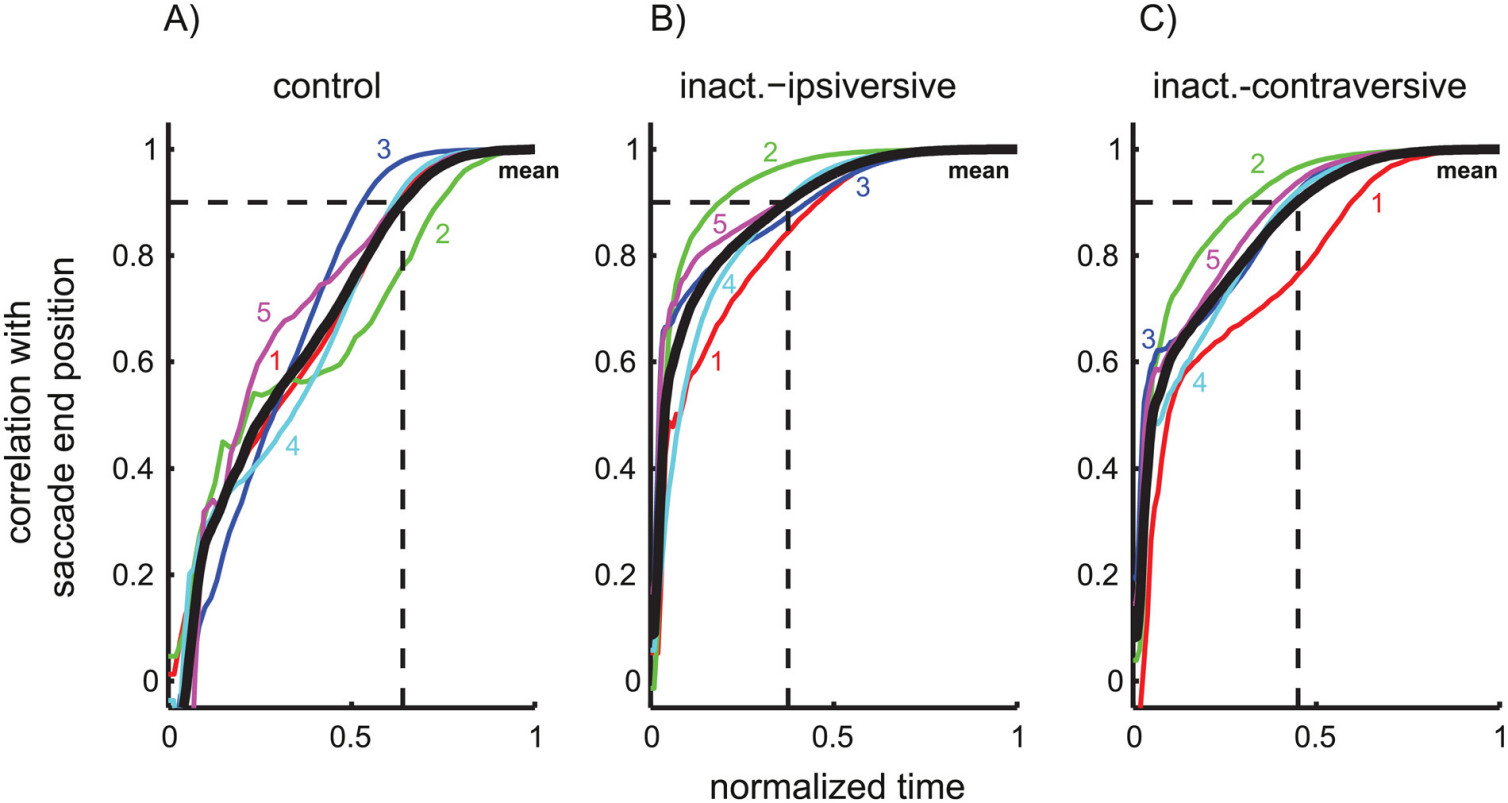
Statistical coupling between eye position during the saccade and eye end position. Pearson’s correlation coefficient between the eye position during the saccade and the saccade end position. Time = 0 indicates the saccade onset and time = 1 the saccade end. Thin lines: correlation time courses for each monkey. Thick solid: average across monkeys. Thick dashed: first increase of the mean correlation above 0.9. In control saccades (A) this increase occurred at 64% of the total saccade duration and later than for saccades during cFN inactivation (B: ipsiversive: 38%; C: contraversive: 45%).

During cFN inactivation (Fig 9B/9C), the correlation of the eye position with saccade end position Eq (2) increased towards a value of 1 faster than during control saccades (Fig 9A). This was quantified by computing, for each monkey and saccade condition, the time *t*_*ρ* = 0.9_ after saccade onset when the correlation trajectory reached a value of 0.9 ($\rho_{ye}\left(t_{\rho =0.9},\;\bar{A}\right)=0.9)$. We expressed this time as a percentage of the respective saccade duration $\bar{D}$. This percentage ($P_{\rho =0.9}=\frac{t_{\rho =0.9}}{\bar{D}}\cdot 100$) was larger for control saccades (63±8%) than for ipsiversive (36±11%) or contraversive (43±10%) saccades during cFN inactivation (ANOVA: F(2,8) = 10.82; p<0.01; Scheffé: p<0.03). The difference of *P*_*ρ* = 0.9_ between ipsi- and contraversive saccades during cFN inactivation did not reach significance (Scheffé: p = 0.55). The faster increase of the correlation trajectory during cFN inactivation shows that the saccade end position depended more strongly on the eye position earlier during the saccade. This means that saccades executed during cFN inactivation are less efficient in online compensating for motor errors that occur early during its time course. Underlying reasons for this effect may be reduced online feedback and increased planning noise.

#### Modeled noise components

**Fig 10:**
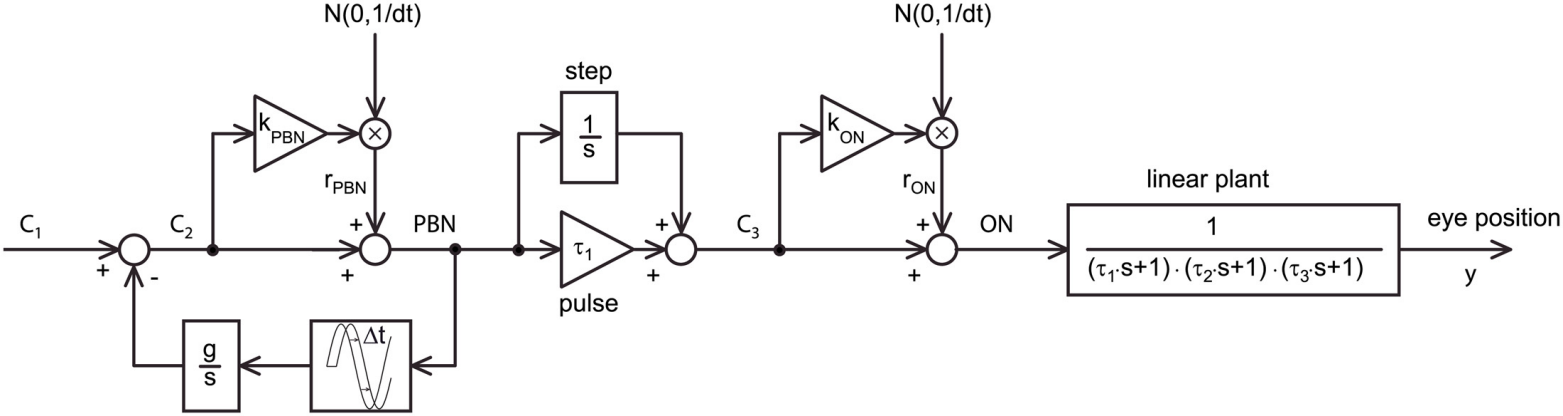
Motor noise and Premotor noise. Model used for simulation of signal-dependent motor noise in the oculomotor neurons (ON) and in premotor burst neurons (PBN). Both of these signals are assumed to be additively contaminated by Gaussian white noise, which is linearly scaled (factors: *k*_*ON*_, *k*_*PBN*_) by the control signals C_3_ and C_2_. The linear plant is a third-order lowpass approximating the recruitment dynamics of the muscle and the overdamped dynamics of the eyeball. The output of this model is the eye position (y). While the noise in the ON is only propagated feedforward, noise in the PBN is assumed to be filtered by a closed feedback loop with a delay of Δt = 4 ms, and an integrator with gain g. The transfer between the premotor burst and the oculomotor neuron is modeled with the classical pulse-step superposition [21].

To understand how the model accounts for the different variance structures of control saccades and saccades during cFN inactivation it is necessary to consider how inactivation affects the different components of noise. During the control condition PBN noise played only a minor role in end point variance. This is because the variance trajectory of signal-dependent noise in the PBN had an increasing-decreasing pattern (Fig 2C) while the variance trajectory of both planning and ON noise increased monotonically (Fig 2B/2D). Similarly, decomposing the simplified model fits of the variance trajectories during cFN inactivation (Fig 4B/4C) into the components related to PBN noise and accumulating noise showed that the feedback gain was still large enough to compensate for this PBN noise at movement end (Fig 5B/5C). The variance of the PBN-component at movement end, expressed as a percentage of its peak, was 30±15% for control saccades (Fig 5A) and 10±11% for ipsiversive saccades during cFN inactivation (Fig 5B). The difference was not significant (paired t-test: p = 0.11). The peak variances of the PBN-noise components (Fig 5) were larger in ipsiversive saccades during cFN inactivation (1.1±0.6 deg^2^) than during the control condition (0.4±0.2 deg^2^), but the difference (0.7 deg^2^) was much smaller than the corresponding effect on end point variance (11.3–1.6 = 9.7 deg^2^, Table 1A/1B).

Thus, inactivation effects on PBN noise did not contribute much to inactivation effects on end point variance (pair wise differences of columns *var*_*Amp*_ between Table 1B and 1A) which were almost entirely explained by the ipsilateral inactivation effects on the components related to accumulating noise: the mean squared difference between these inactivation effects was 0.086 deg^4^ and amounted to only 0.07% of the mean squared inactivation effects of the total observed end point variance.

The increase in accumulating noise of ipsiversive saccades during cFN inactivation was due to two different factors, the relative contribution of which differed between monkeys: first, the hypermetria of ipsiversive saccades during cFN inactivation (prominent in monkeys 3, 4, and 5, see Table 3A/3B, column *Amplitude*) and, second, the increased accumulating-noise coefficient *k*_*A*_ (prominent in monkeys 2 and 5, see Table 2A/2B, column *k*_*A*_). Correspondingly, monkey 5, who was strongly affected by both factors, also showed the strongest increase in end point variance (Table 1A/1B, column *var*_*Amp*_).

## Discussion

The current study quantified time courses of inter-trial variability of eye position during saccades. The variance/covariance structure of this variability was modeled by superposition of different noise sources. Signal-dependent noise entering within a premotor feedback loop (PBN noise) was demonstrated to affect the variance/covariance structure of saccades in a very specific way that provides a new approach to disentangle feedback-controlled motor noise from accumulating noise (i.e. planning and ON noise). Simulations showed that PBN noise increases the variance trajectory during the saccade but plays a minor role for end point variance which is dominated by accumulating noise. Inactivating the cFN on one side of the cerebellum strongly increased the end point variance of ipsiversive but not contraversive saccades. For ipsiversive saccades, inactivation effects were also observed in the variance/covariance trajectories, reflected by a systematic decrease of the feedback gain of the fitted noise model. This decrease of the feedback gain, though significant, was too small to explain the increase of the ipsilateral end point variance. The proposed model explained this increase of end point variance by an increase of planning and ON noise during cFN inactivation.

### Signal dependency of noise sources

All three signal-dependent noise sources used in our model were assumed to show a variance Eq (12), or power densities (Eqs 14/18) proportional to the expectation of the squared mean signal. For ON noise this signal dependency was adopted from previous studies [2, 3]. The study of Jones et al. [32] suggested that this type of signal dependency is closely related to the stochastic nature of muscle unit recruitment, and that force variability during voluntary isometric muscle contraction is "… independent of presynaptic noise in the motor command." So far it is not known whether this conclusion generalizes from tonic muscle activation to the fast changing dynamic changes occurring during saccades. Our findings provide a first hint that the variance trajectory of saccades may not be explained by signal-dependent noise in the ON alone but is also subject to signal-dependent noise entering within an internal feedback loop (PBN noise). Even though this noise component was relatively small compared to "accumulating noise", it significantly improved the model fit (Fig 3).

For the PBN noise, the assumption that power density increases with increasing firing rate of the PBN, is generally supported by basic statistics of neuronal firing rates. When these statistics are modeled by Poisson statistics, the variance of the firing rate increases linearly with its expectation [33]. Our assumption Eq (18) deviates from that model since we assumed a quadratic rather than linear scaling of the power density. However, it is very difficult to judge whether the noise within the feedback loop follows the Poisson statistics or not, especially since the physiological origin of so called PBN noise is not known (see the discussion of the cFN-inactivation effects on the feedback gain below). Thus, the assumption expressed in Eq 18 must be considered a somewhat arbitrary but not implausible first approach.

The assumption that planning noise is signal dependent Eq (12) is based on the consideration that the precision of the planned saccade amplitude relies on the precision of sensory signals, target selection, and the motor plan. These signals are believed to be represented in spatiotopic maps the resolution of which decrease with increasing spatial eccentricity. This has been shown for visual target representations in cortical areas [34] as well as for representations of saccadic motor plan in the superior colliculus (SC) [15]. Consequently, a constant precision of a target location in such a map translates in a spatial uncertainty which increases with increasing target eccentricity. Assuming that the planned saccade amplitude is transformed by the motor system into the amplitude of the executed saccade with a certain gain factor (which does not necessarily equal one) leads directly to an approximation of the signal dependency of planning noise characterized by a constant coefficient of variation Eq (12) (see S1 Text). For target eccentricities about 10 deg used in the current study Eq 12 it is a very close approximation to models of planning noise [16] that include a coefficient of variation that depends on eccentricity. Notably, our result that the variance trajectory predicted by planning noise differs qualitatively from that of PBN noise (Fig 2B/2C) does not depend on whether the coefficient of variance of planning noise is assumed to be constant or not. This can be seen from Eq 10 which shows that the shape of the variance trajectory predicted by planning noise depends only on the planned saccade trajectory $\bar{y}\left(t,\;A\right)$ and not on assumptions concerning the signal dependency of the variance ($\sigma_{A}^{2}$) of the planned saccade amplitude. Thus, these assumptions have no impact on our main conclusion that PBN noise contributes to the variance of eye position during the saccades.

### Inter-trial variance of control saccades

The analysis of the relation between amplitude and duration for a group of saccades with selected initial motor errors (10±2.5 deg) showed that the variance of the saccade amplitude *var*_*Amp*_ was smaller than the lower limit *α*^2^∙*var*_*TDur*_ expected under the assumption that all variations of amplitude and duration are explained by variations along the main sequence. This finding is a reproduction of results of Jürgens et al. [11] who also underlined that, for saccades with identical initial motor errors, saccade amplitudes are remarkably stable despite considerable variation in saccade duration. Whether the *var*_*Amp*_ we observed was smaller than expected depends critically on the correct estimation of main sequence slope $\alpha =\frac{\Delta Amp}{\Delta Tdur}$. In our monkeys, *α* was 0.66±0.08 deg/ms, and smaller than the value given by Fuchs (1967, *α* = 1 deg/ms), but still larger than in humans (Becker [22]: *α* = 0.4 deg/ms; Jürgens et al.[11]: *α* = 0.2 deg/ms). Thus, our conclusion that, for a given initial motor error, saccade amplitude is stabilized against variations of saccade duration, is justified and reproduces a well-known argument for the existence of internal feedback in the saccade system. Other arguments for such a feedback were derived from analyses [13] showing that saccade amplitude is partially compensated for variation in saccade peak velocity. Quaia et al. [13] observed in three monkeys and for the motor error of 10 deg a "percent compensation for speed" of *C*_*S*_ = 53±15%; N = 3. The percent compensation for speed we obtained by repeating the very same analysis with our data (*C*_*S*_ = 54±18%; N = 5) did not differ from their results. However, as underlined by Quaia et al. [13], the mechanisms underlying such a partial compensation are not yet completely understood.

The current study extends previous findings by showing that such partial compensation of saccade amplitude for variation in other saccade parameters can be explained by internal feedback of PBN activity that is contaminated by signal-dependent noise. In contrast to previous studies, we did not restrict our observation to just two saccade parameters (e.g.: amplitude and peak velocity) but considered the variance/covariance structure of the entire saccade trajectory. Thus, the empirical basis of the current study constitutes a more comprehensive description of the statistic process of saccade trajectories. The variance increase during the saccade showed single or double S-shapes (Fig 2A). In two animals, the variance reached a local maximum during the saccade. These empirical variance trajectories were compared with those predicted by three potential noise sources: variance of the planned saccade amplitude (1: planning noise) and signal-dependent motor noise entering at the input of an oculomotor plant (2: ON noise) or within in a premotor feedback loop (3: PBN noise). Both planning noise and ON noise induce monotonically increasing variance trajectories whereas PBN noise entering within an internal feedback loop induces increasing-decreasing variance trajectories (Fig 2B/2C/2D). This important difference between noise entering within or downstream from a premotor feedback loop does not depend critically on the fine tuning of the particular model we used in our simulations. It holds for a large class of plant models and feedback models. The supplementary information (S3 Text) shows that this model class can be specified without referring to any particular parameterization of the plant. The model comparison revealed that superposition of PBN noise with accumulating noise (planning and ON noise) could explain the measured variance and covariance trajectory (Fig 4A/4D). The three model parameters [*k*_*A*_, *k*_*PBN*_, *g*] of this simplified model explained not only the inter-trial trajectory noise as represented by a population estimate but also that of each individual monkey (Table 2A, column R^2^).

The explanatory power of this simplified model proved equal to the full model and superior to a model that included planning noise and ON noise but not PBN noise (Fig 3). We may infer two things from this. First, the predictions of the effects of planning-noise and ON-noise did not differ enough from one another to be unambiguously separated using the variance and covariance trajectories that we observed. Second, the significant contribution of the PBN noise suggests that a part of the observed trajectory noise is due to signal-dependent noise entering within an internal feedback loop. The proposed model fit offers an empirical estimate of the gain of this feedback (*g* = 240±59 s^-1^, Table 2A) whereas the gain values used in the literature so far (Dean [35]: *g* = 110 s^-1^; Van Opstal and Goossens [15]: *g* = 80 s^-1^) were not derived from experimental data.

Even though the observed variance and covariance trajectories suggest that signal-dependent noise entering within an internal feedback loop contributes significantly to the variance of eye position during the saccade, this noise component accounted only for a small proportion of the observed end point variance. End point variance was dominated by accumulating noise entering before or after the internal feedback loop. It is important to note that this efficiency of noise compensation achieved by premotor feedback explains why PBN noise can be ignored for minimizing end point variance. Thus, the current study supports the crucial role of ON noise for minimizing the variance of eye position at and shortly after movement end as put forward by Harris and Wolpert [2, 3].

In a previous study [16] van Beers presented another approach to explain observed saccade variability as superposition of different variance components, namely *sensory noise* and three types of motor noise, *signal dependent noise*, *constant noise*, and *temporal noise*. Comparing these components with those of the current study, one can identify van Beers' *sensory noise* and *signal dependent noise* with our planning noise and ON noise, respectively. Like our results, van Beers [16] could not estimate the relative contribution of *sensory noise* and *signal dependent motor noise* from the movement data. Based on literature on perceptual localization [36] he estimated that *sensory noise* accounted for about 57% of end point variance. Since our PBN noise was almost irrelevant for end point variance, the very same estimate can be applied to our data (resulting in planning variance accounting for 32% of end point variance). However, as already noted by van Beers [16], it is questionable whether estimates of perceptual *sensory noise* can substitute estimates of the variance in planned saccade amplitude. Therefore we did not further pursue this approach and used the term *planning noise* rater than *sensory noise*.

Unlike van Beers [16] we did not consider *constant motor noise* (i.e. signal independent noise in the ON). This simplification is due to the limitation of our dataset to a small range of target eccentricities (10±2.5 deg). For such a dataset, model fitting does not substantially benefit from adding constant motor noise. Thus, ignoring constant motor noise was an appropriate simplification for our study, but does not argue against the concept of *constant motor noise* per se. Adding *constant noise* in our model of ON noise would not have improved its ability to explain the observed double S-shaped of the variance trajectories (Fig 2A) because the variance trajectory resulting from constant motor noise increases monotonically (as it can be shown by evaluating Eq 15 with *Q*_*ON*_(*τ*) = *const*.). Therefore, not accounting for a potential *constant motor noise* does not compromise our conclusion that ON noise entering downstream from a premotor feedback loop alone is not sufficient to explain the time course of the variance of eye position during the saccade.

The proposed method is of potential interest for investigating the role of online feedback in other data sets. For example, it has been hypothesized that online feedback is impaired in saccades elicited by electrical stimulation of the SC [37]. Fitting our noise model to the variance/covariance trajectories of these saccades would provide an empirical estimate of the feedback gain under these conditions and could contribute to testing of this hypothesis.

### Effects of cFN inactivation on inter-trial variability of saccades

#### Decreased feedback gain

Inactivating the cFN on one side decreases the estimated feedback gain for ipsiversive saccades. This indicates that, under control conditions, cFN activity aids local feedback in the saccade system. This conclusion is compatible with previous suggestions [24–26, 35], but in addition to these studies, the current analysis also provides a quantitative estimate of the size of the cFN contribution to the internal feedback. During cFN inactivation, the feedback gain for ipsiversive saccades decreased to about 39% (= 94/240) of its value during the control condition (Table 2A/2B). The feedback remaining after inactivation is unlikely to reflect residual cFN activity for three reasons. First, the muscimol injections certainly reached the target cFN since all monkeys showed clear inactivation effects on saccade trajectory and gain (Table 3). Second, the injection probably affected all saccade-related neurons in the cFN. According to Robinson et al. [17], a muscimol injection of 4 μl did not inactivate more cFN neurons than did a 1 μl injection at the same location. Third, GABAergic inhibition of the cFN by the cerebellar Purkinje cells is the only mechanism by which cerebellar feedback is transmitted to the cFN [38]. Thus, our finding seems to indicate that even complete cFN inactivation does not entirely abolish internal feedback control in the saccade system. Feedback based on neural substrates other than the cFN was also suggested by studies [30, 39] showing that cFN inactivation in the cat does not prevent intra-saccadic compensation of perturbations induced by electrical stimulation of the SC. Many primate studies on saccade feedback control focus on brainstem-cerebellar loops [24, 26, 35]. However, primate experiments with electrical stimulation of the SC [40, 41], or recordings of SC activity during saccades that were artificially slowed by muscimol injections in the omnipause neurons [42] suggested the existence of a feedback loop including the SC. In the monkey, it is not known at present whether the cFN is involved in such a feedback loop across the SC. The method for estimating the feedback gain that we used here does not make assumptions about the actual physiological substrate of the feedback or the so-called "premotor burst neuron" (PBN) and does not allow any corresponding inference. Signal-dependent noise entering within an integrating loop transfer function would cause the same characteristic increasing-decreasing pattern of the variance trajectory (Figs 2C and 5A/5B/5C), independent of whether the feedback was provided by a single or by multiple parallel loops.

#### Increased end point variance

The model explained the increased end point variance of saccades ipsiversive to an inactivated cFN not by the decreased feedback gain (Fig 5A/5B) but by increased accumulating noise (planning and ON noise). This increase was due to two different factors. The first factor was the signal dependence of the accumulating noise Eq (12) which explains why end point variance was increased for the hypermetric ipsiversive saccades but not for the hypometric contraversive saccades. Both planning and ON noise may contribute to this factor since both show the same type of signal dependency (Eqs 12/14). The second factor was the inactivation effect on *k*_*A*_ which was observed in four of five monkeys (justified by the model comparison showing that these effects contributed significantly to model performance during cFN inactivation). This second factor indicates that, for ipsiversive saccades, cFN inactivation has an amplifying effect on the signal dependence (*k*_*A*_) of accumulating noise.

Concerning the two potential components of accumulating noise, such an amplifying effect seems unlikely for the signal dependency of ON noise because it is believed to be determined by the mechanism of muscle recruitment [32], and because this mechanism should not depend on how much of the excitation of the ON is provided by the cFN.

The alternative explanation is that cFN inactivation affects the signal dependence of planning noise. For motor plans represented by the active position on a spatiotopic map, the planning-noise coefficient *k*_*A*_ is proportional to the inter-trial standard deviation of the motor plan (see S1 Text). Therefore, this alternative explanation would suggest that the inter-trial variability of internal representations of the motor plan would be affected by cFN inactivation, and implies that such effects occur upstream from the brainstem pulse generator. This is compatible with the suggestion of Pélisson et al. [30] that the saccade dysmetria induced by cFN inactivation is caused by projections of the cFN, not only to the immediate premotor centers of the reticular formation, but also to the SC. Our finding shows that most of the additional saccade variability during cFN inactivation accumulated monotonically during the saccade and could not be explained by noise entering within a feedback loop with reduced gain. This finding indicates that the variability of the neural signals driving the feedback loop is most probably affected by cFN inactivation.

In summary, effects of cFN inactivation on the variance/covariance structure can be modeled by two different mechanisms: 1) a gain decrease of internal feedback and 2) an increase of accumulating noise entering in a feedforward path outside of the feedback loop. Both of these effects help to explain why the correlation trajectory increased faster during cFN inactivation than under control conditions (Fig 9). Both reduced feedback gain and increased accumulating noise enlarge noise that is not compensated until the end of the saccade. These noise sources thereby increase the correlation of the eye position during the saccade with eye end position. Saccades executed during cFN inactivation were more variable across trials but show stronger statistical coupling across time within a trial.

### Differences of cFN inactivation effects between monkeys

The ipsilateral inactivation effects on the fitted feedback gain (Fig 8C, left bar, Δ*g*) did not correlate across monkeys with the effects on the noise coefficient of the accumulating noise (Fig 8A, left bar, Δ*k*_*A*_). This lack of correlation indicates that effects of cFN inactivation on the feedback gain and on the accumulating noise depend on different mechanisms which were differently affected in different monkeys. It shows that cFN inactivation changes the variance/covariance structure in a complex way varying in at least two degrees of freedom. The factors underlying these idiosyncratic differences of cFN inactivations are not known, but they further support the notion of differential effects of the cFN on circuits inside and outside (possibly upstream) of an internal feedback loop. Further support of this hypothesis is provided by a similar dissociation between the inactivation effects of saccade gain and saccade dynamics (Fig 7A). This finding reproduces in the monkey an observation which was previously reported in the cat[43].

Taken together, these results suggest that cFN inactivation effects on the saccade are mediated by (at least) two mechanisms. First, a contribution of cFN activity in online feedback which is relative consistent across individuals and second, an effect of cFN inactivation on feedforward control which is more variable across individuals. The inter-individual consistence of the effect on the feedback mechanisms (i.e. the reduced feedback gain) indicates that this effect is due to a direct contribution of the (missing) cFN activity in an online feedback mechanisms. The inter-individual inconsistence of effects on feedforward control (i.e. components of saccade dysmetria due to altered planning and the increase of accumulating noise) indicates that these effects comprise (besides primary lesion effects) also adaptive modifications of saccade planning. In an acute cerebellar lesion it is not surprising that such secondary effects on compensatory strategies dissociate across individuals. This interpretation is compatible with the idea that saccade adaptation is achieved by modifications of both feedback and/or feedforward mechanisms[44]. It is also compatible with the more general concept of cerebellar function being involved in both feedback and feedforward mechanisms [19]. A particular new aspect demonstrated by the current study is that dissociations between feedback and feedforward mechanisms of saccade control may not only be observed in changes of the mean trajectory of the saccade [44] but also in changes of the structure of its variance (as represented by the inter-subject inconsistency of Δ*k*_*A*_ compared to the consistency of Δ*g*, Fig 8A/8C). For two reasons this interpretation does not imply that absolute changes in saccade gain should correlate with changes in planning noise across individuals. First, because gain changes may reflect combined modifications of feedforward and of feedback mechanisms and, second, because mean and variance of planning may change independently of each other since both represent different aspects of a feedforward control mechanism. Also our data do not support a dependency between effects on saccade gain and planning noise because the two monkeys with the smallest and the largest gain change (#1 and #5, respectively, Fig 7A) were not identical to those with the smallest and largest change in the coefficient of variation of planning noise (#3 and #2, respectively, Fig 8A).

### Conclusion

The current study shows that the variability of saccade trajectories is not completely explained by a superposition of planning noise and motor noise entering at the level of the oculomotor neurons (ON noise). During the saccade but not at saccade end, additional motor noise entering within an internal feedback loop contributes significantly to the variance/covariance structure of saccade trajectories. Fitting the observed variance/covariance trajectories with a model that uses only three free parameters provides a new approach to estimate the strength of the internal feedback.

Effects of inactivating the cFN on the saccade variability were most prominent for saccades ipsiversive to the inactivated cFN. These saccades exhibited an increase of end point variance and a stronger statistical coupling between eye position during the saccade and saccade end position. The proposed noise model explained these effects by a decrease of the gain of the internal feedback loop and by an increase of planning and ON noise. The decrease of the fitted feedback gain supports the role of the cerebellum in an internal feedback acting through its connections to the brainstem. The increase of planning and ON noise was partly induced indirectly by the hypermetria of ipsiversive saccades, because the signal dependency of both noise types amplified their effects on the variance/covariance of the saccade trajectory. In addition to this effect, cFN inactivation seems to cause an increase of planning- and ON noise components of ipsiversive saccades in some individuals.

## Methods

### Data acquisition

Eye movements were recorded in five adolescent rhesus monkeys trained to track a stepping laser target. Parts of these data and the methods have been published previously [17, 45]. Briefly, monkeys were trained to make accurate saccades to a small laser target (diameter 0.3 deg) controlled by a mirror galvanometer (G120D, General Scanning, Watertown, MA, USA). Eye position was monitored with the search coil technique [46]. Eye position and target position were recorded on magnetic tape and digitized off-line at 1 kHz and filtered offline with a symmetric (zero-phase) Gaussian lowpass (3dB attenuation at 100Hz). The laser target stepped between target positions in the range of ±20 deg eccentricity on the horizontal meridian. The target step amplitudes ranged from 5 to 20 degrees of visual angle. The monkey tracked the target with saccades while its head was immobilized. Eye movements were recorded before and after injection of about 1 to 1.5 μl muscimol (solution: 1mg/ml in normal saline) into the cFN on one side of the cerebellum. The injection of this GABA_A_ agonist causes a functional, reversible inactivation of the cFN [17]. All data analyzed for each monkey (pre- and post-injection) originate from the same recording session. The Institutional Animal Care and Use Committee of the University of Washington evaluated and approved all procedures for this research.

### Data analysis

All numerical computations, simulations and optimizations were executed using MATLAB^®^ (The MathWorks, Inc., Natick, Massachusetts, USA).

For each monkey, offset and gain of a linear calibration function was established to transform the horizontal coil raw signal to horizontal eye position. Offset and gain were fitted to minimize the mean squared error between target and eye position during stable fixation periods (after execution of potential corrective saccades) during the pre-injection measurements. This linear calibration was then applied to eye movement data both pre- and post-injection. The calibrated eye position during the saccade was expressed relative to the eye position at saccade onset and mirrored for monkeys with muscimol injections in the left cFN so that positive saccade trajectories correspond to movements directed ipsilaterally to the lesion side. The beginning and the end of saccades were defined by the times when eye velocity rose above or fell below 10% of peak velocity. For each saccade the initial motor error was defined by the retinal target eccentricity at the time of saccade onset.

#### Mean saccade trajectory

The mean saccade trajectory, averaged across trials, was parameterized with respect to two particular aspects: 1) the shape of the velocity profile of saccades with 10 deg amplitude, quantified by total saccade duration, peak velocity and the duration of the deceleration phase (i.e. the time between peak velocity and saccade end), and 2) the amplitude of saccades with 10 deg initial motor error. Based on these parameters, inactivation effects on the velocity profile of 10 deg saccades were quantified by the signed differences ΔVP = [Δpeak velocity, Δtotal dur.,Δdeceleration dur.] between saccades during cFN inactivation and control saccades. The effect of pharmacological inactivation of the cFN on the saccade gain was quantified by the corresponding differences (Δamplitude) of the amplitude of saccades with 10 deg motor error. All effect sizes were computed separately for ipsiversive and contraversive saccades. This evaluation differs from previous studies in that effects of cFN inactivation on peak velocity, total duration, and deceleration duration were mostly reported for averages across saccades responding to the same target step size or across saccades with the same motor error. In contrast, the current study evaluates these parameters for fixed saccade amplitude. The advantage of this method is that effects on the main sequence can be quantified independently of effects on the saccade amplitude. To avoid problems related to differences of the distributions of motor error and saccade amplitude between different monkeys and different saccade conditions (control, ipsiversive inactivated, and contraversive inactivated) the relations between amplitude, peak velocity, and skewness [6] were fitted separately for each monkey and each saccade condition. Total saccade duration, peak velocity and the duration of the deceleration phase of saccades with 10 deg amplitude were then determined by interpolation in these fits.

If the neural activity of the cFN and its effect on the mean saccade trajectory under control conditions were identical in all monkeys, then we would expect differences of cFN inactivation effects between monkeys being determined by a single degree of freedom, i.e., the strength of attenuation of cFN activity achieved by the muscimol injection. In that case, and if inactivation effects on the mean saccade trajectory can be approximated as being proportional to the strength of cFN attenuation, the different aspects of inactivation effects on the mean saccade trajectory would be proportional to each other. To test this, we investigated whether the components of ΔVP and Δamplitude were proportional to each other, 1) for the three components of ΔVP only and 2) for a total effect vector [ΔVP; Δamplitude] combining all effect aspects. The proportionality was quantified by the fraction of total variance covered by the projection of the normalized data on the constrained major axis (i.e., on the connecting line between the mean and the coordinate origin). A value of this fraction (*F*_*prop*_) of 1 indicates that the different effect components were perfectly proportional to each other. For *n* spherically distributed effect components (i.e., no support of proportionality between the components) *F*_*prop*_ takes the value of 1/*n*. Therefore, we defined the proportionality index *I*_*prop*_ by

$$I_{Prop}=\frac{F_{prop}-1/n}{1-1/n}\;;\;I_{prop}\leq 1$$ (1)

To analyze the proportionality of the components of ΔVP or of the total effect vector [ΔVP; Δamplitude], *n* was set to 3 or 4 respectively.

#### Inter-trial variability of saccade trajectories

The inter-trial variability of saccade trajectories was evaluated by sorting all saccades into 8 different classes of horizontal motor errors, centered on ± 5, 10, 15, 20 deg and each with a width of ±2.5 deg. Since in all of the 5 monkeys most saccades (ranging between 397 and 1410 saccades per monkey) fell in the class centered on the motor error of 10 deg, only these saccades were further analyzed. The standard deviation of the motor error within this class was 1.00±0.47 deg, averaged across all monkeys and saccade conditions. Separately for each monkey, and for each saccade condition, we computed the mean saccade amplitude ($\bar{A}$), normalized all saccade trajectories in time on the mean saccade duration ($\bar{D}$), and re-sampled them to 100 equidistant samples on the time axis $t\in \left[0,\;\bar{D}\right]$. The normalized and re-sampled eye position traces are indicated by *y*(*t*). For each of these sampling times the following three dependent measures were computed by averaging across all trials of the class: 1) the mean eye position, 2) the variance of the eye position, and 3) the covariance of the eye position at this sampling time with the saccade end position. The three resulting time courses will be called the 1) main-sequence trajectory ($\bar{y}\left(t,\;\bar{A}\right)$), 2) variance trajectory (${var}_{y}(t,\;\overset{-}{A})$), and 3) covariance trajectory (${cov}_{ye}(t,\;\overset{-}{A})$). The main-sequence trajectory $\overset{-}{y}(t,\;\overset{-}{A})$ is an estimate of the saccade trajectory which would be executed for a planned amplitude $\overset{-}{A}$ in the absence of any motor noise. In that case the executed and the planned amplitudes would be identical, i.e. $\overset{-}{A}\;=\;\overset{-}{y}(\overset{-}{D},\;\overset{-}{A})$. The variance trajectory ${var}_{y}(t,\;\overset{-}{A})$ describes the increase of variability of the eye position during the saccade, whereas the covariance trajectory ${cov}_{ye}(t,\;\overset{-}{A})$ provides the additional information necessary to evaluate Pearson’s correlation coefficient $\rho_{ye}(t,\;\overset{-}{A}\;)$, which quantifies (for normally distributed eye positions) the statistical dependency between the eye position at time *t* and saccade end.

$$\rho_{ye}\left(t,\;\bar{A}\;\right)=\frac{cov_{ye}\left(t,\;\bar{A}\right)}{\sqrt{var_{y}\left(t,\;\bar{A}\right)\cdot var_{y}\left(\bar{D},\;\bar{A}\right)}}$$ (2)

The time course of this correlation (which we call the correlation trajectory) approaches 1 at the end of the saccade and is an important indicator of rapid movement control occurring during movement execution [47]. Fast increase of the correlation trajectory towards one indicates that early deviations of the eye position from the main sequence trajectory are highly predictive for the saccade end position, whereas slow increase of the correlation trajectory indicates that late components of movement control can overwrite the effects of early deviations. It is important to note that such late control commands do not necessarily reflect compensatory online feedback but can also result from non-compensated motor noise. Non-compensated motor noise that occurs during the saccade causes later variability which is independent of earlier variability and therefore decouples early and late variability. Compensatory feedback decouples early and late variability even further because it prevents the propagation of early to late variability.

### Modeling of the saccade-generator

The main sequence trajectory was modeled as the mean response of a linear saccade generator shown in Fig 10. The dynamic system transforming the activity of the oculomotor neurons (ON) to eye position was modeled by a third-order low-pass with the same three time constants (*τ*_1_ = 223, *τ*_2_ = 14, *τ*_3_ = 4 ms) as used by previous studies [2, 3, 8]. The two shortest mechanical time constants (*τ*_1,_ *τ*_2_)used in these studies where estimated for the human oculomotor plant and differ from those in the rhesus macaque (*τ*_1_ = 100, *τ*_2_ = 10)[48], *τ*_1_ = 104, *τ*_2_ = 23[49]). However, these differences are not critical for the conclusions of the current study (see S3 Text). The dynamics to transform the activity of the premotor burst neuron (PBN) into the activity of the oculomotor neurons were described by the classical pulse-step superposition [21], and the PBN was assumed to be enclosed in an internal feedback loop with a loop-transfer function composed of a delay and an integrator. This principle scheme of an internal feedback is a common element of many models in the literature [10, 11, 14, 15]. The numerical value of the feedback delay (4 ms) was also adopted from the literature [11, 15, 35]. These different models do not all agree about whether the integrator is located in the feedback- [11, 14] or in the feedforward-branch [15] of the internal loop. Fig 10 shows the scheme of the former models in which the driving input is step-like. In contrast, to correspond with the feedforward-version, the driving input *C*_1_ should be pulse-like, and should be added to the input rather than to the output of the integrator. However, these differences have no relevance for the dynamic transfer of the signal-dependent noise *r*_*PBN*_(*t*) to the output of the loop, because this is determined by the loop transfer function (i.e., the product of feedforward and feedback transfer functions within the loop) which is similar in all of the mentioned models. As in previous model formulations of the brainstem saccade generator [21], the firing rates of ON and PBN were expressed in units of eye position and eye velocity respectively.

To obtain for each monkey and for each saccade condition estimates of the internal control signals ${\overset{-}{C}}_{2}(t)$, and ${\overset{-}{C}}_{3}(t)$ (Fig 10) the model was inverted and applied on the observed main-sequence trajectory $\overset{-}{y}(t,\;\overset{-}{A})$. The inversion was performed by computing numerical differentiation of the main-sequence trajectory and using

$${\bar{C}}_{2}\left(t\right)=\tau_{2}\;\tau_{3}\;\dddot{\bar{y}}\left(t,\;\bar{A}\right)+\;\left(\tau_{2}+\tau_{3}\right)\ddot{\bar{y}}\left(t,\;\bar{A}\right)+\;\dot{\bar{y}}\left(t,\;\bar{A}\right),$$ (3)

and

$${\bar{C}}_{3}\left(t\right)=\tau_{1}\;{\bar{C}}_{2}\left(t\right)+\;\int_{\upsilon =0}^{t}{\bar{C}}_{2}\left(\upsilon \right)\;d\upsilon .$$ (4)

**Fig 11:**
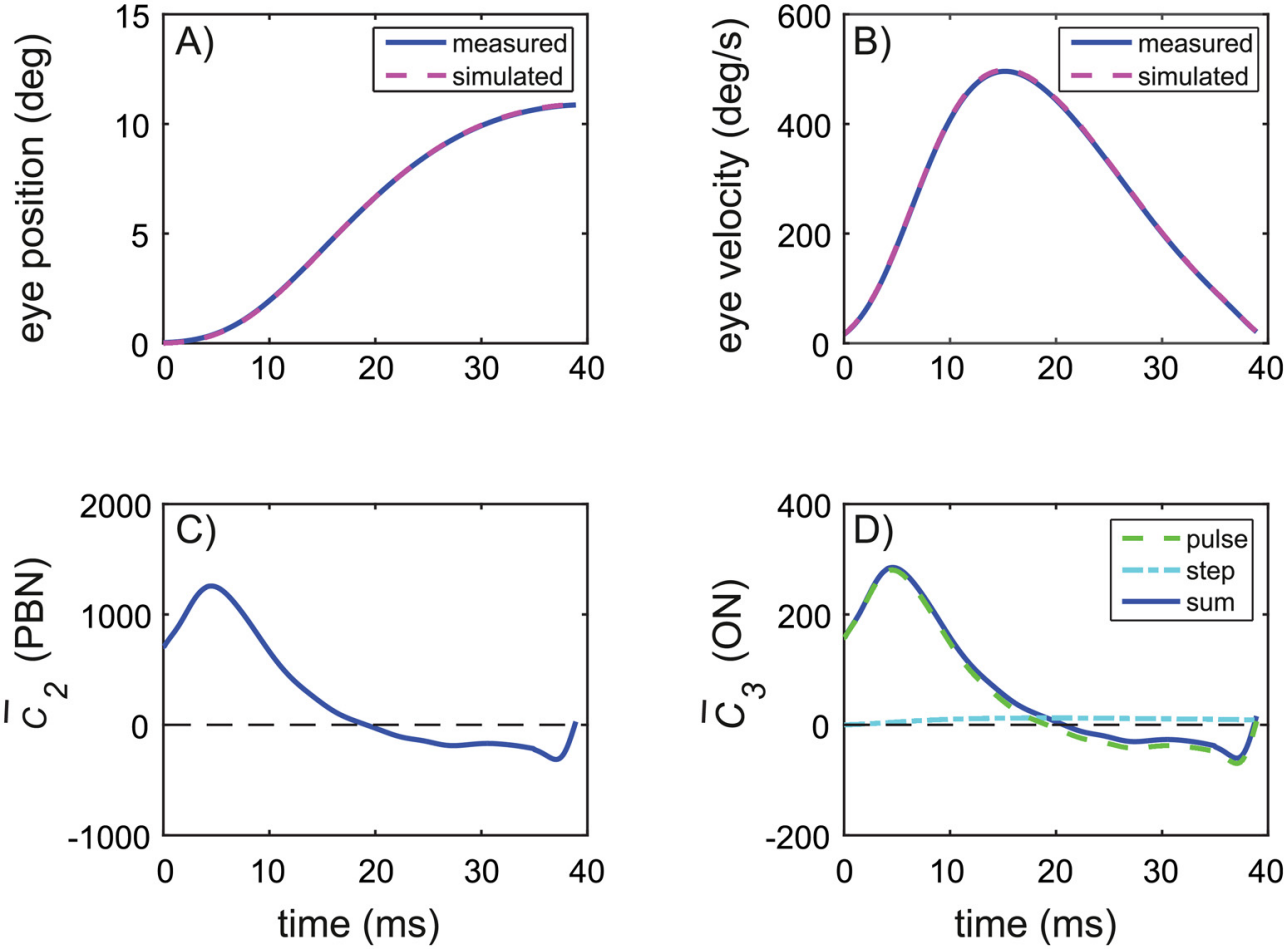
Identification of internal control signals. A, blue: main-sequence trajectory $\overset{-}{y}(t,\;\overset{-}{A})$ of a saccade with 10 deg motor error of one monkey. B, blue: The corresponding mean velocity trace. C/D: Control signals ${\overset{-}{C}}_{2}$ and ${\overset{-}{C}}_{3}$ representing the mean activity profile of the premotor burst neuron (PBN) and the oculomotor neurons (ON) respectively. These signals were computed by inverting the corresponding linear systems depicted in Fig 10 (see Eqs 3 and 4) and applying them to the main-sequence trajectory (A). A, magenta: The simulated output of the (non-inverted) linear system Eq (5) driven by ${\overset{-}{C}}_{2}$. B, magenta: The derivative of this simulated output. The matching of dashed and solid lines in C/D verifies accurate system inversion.

Here, the first summand of Eq 4 represents the pulse-, and the second summand of Eq 4 represents the step-component of Robinson’s pulse-step superposition[21]. Fig 11 shows an example of this inversion in the control saccades (Fig 11A/11B, solid) of a typical monkey. The resulting mean activity of the ON (Fig 11D, solid) seems to be almost proportional to that of the premotor burst neuron because the pulse-component (Fig 11D, dashed) is large compared to the amplitude of the step component (Fig 11D, dash-dotted). The inversion was verified by using the MATLAB function lsim to simulate the transfer of ${\overset{-}{C}}_{2}(t)$ through the linear dynamics

$$\frac{Y\left(s\right)}{{\bar{C}}_{2}\left(s\right)}=\frac{\tau_{1}+\frac{1}{s}}{\left(\tau_{1}\cdot s+1\right)\cdot \left(\tau_{2}\cdot s+1\right)\cdot \left(\tau_{3}\cdot s+1\right)}=\frac{1}{s\cdot \left(\tau_{2}\cdot s+1\right)\cdot \left(\tau_{3}\cdot s+1\right)}$$ (5)

and comparing the resulting eye position trace (Fig 11A, dashed) with the observed main-sequence trajectory (Fig 11A, solid).

### Modeling noise of the saccade trajectory

In order to explain the experimentally observed inter-trial variability, quantified by the variance and covariance trajectories, we assumed three independent sources of variability: 1) planning noise resulting from a trial-to-trial variability of the planned saccade amplitude, 2) signal-dependent noise added to the mean activity of the ONs (motor noise), and 3) signal-dependent noise added to the mean activity of the PBNs (premotor noise). The independence of these three noise sources allows their effects on the variability of the saccade trajectory to be analyzed separately. In the following, the computation of the covariance and variance trajectories is described for each source.

#### Planning noise

Saccades differ from goal-directed arm movements in that the mean kinematic parameters of the saccades such as peak velocity, duration, and skewness are more strongly coupled with the saccade amplitude [5, 6]. Thus, in contrast to arm movements which can deliberately be executed at different movement speeds, the average trajectory of saccades (the so-called main-sequence trajectory) is predominately determined by its amplitude. Therefore, saccade planning may be approximated, in a first approach, by the planning of saccade amplitude, and, correspondingly, we define the “planning noise” of the saccade trajectory as the component of its variability that is due to the variability of the planned saccade amplitude (in the absence of any other noise sources). All the saccades in such a hypothetical sample without motor noise would exactly obey the main sequence, and therefore both the variance trajectory *var*_*y*_(*t*) and the covariance trajectory *cov*_*ye*_(*t*) induced by planning noise are determined by only two factors: 1) the variance of the planned saccade amplitude, and 2) the dependence of the main-sequence trajectory $\overset{-}{y}(t,\;\overset{-}{A})$ on the mean saccade amplitude ($\overset{-}{A}$):

$$cov_{ye}\left(t,\;\bar{A}\right)=E_{A}\left\{\left(y\left(t,A\right)-\bar{y}\left(t,\;\bar{A}\right)\right)\cdot \left(A-\bar{A}\right)\right\}$$ (6)

$$var_{y}\left(t,\;\bar{A}\right)=E_{A}\left\{{\left(y\left(t,A\right)-\bar{y}\left(t,\;\bar{A}\right)\right)}^{2}\right\}$$ (7)

In the absence of any motor noise, the eye position *y*(*t*) is identical to the main sequence trajectory $\overset{-}{y}(t,\;A)$. For small differences $A-\overset{-}{A}$, the difference between the actual trajectory and its mean can be linearization:

$$\begin{array}{c} y\left(t,\;A\right)-\bar{y}\left(t,\;\bar{A}\right)=\bar{y}\left(t,\;A\right)-\bar{y}\left(t,\;\bar{A}\right)\\ \approx {\frac{\partial }{\partial A\;}\bar{y\;}\left(t,\;A\right)|}_{A=\bar{A}}\cdot \left(A-\bar{A}\right)\\ =\frac{\partial }{\partial \bar{A}\;}\bar{y\;}\left(t,\;\bar{A}\right)\cdot \left(A-\bar{A}\right) \end{array}$$ (8)

Therefore, Eqs 6 and 7 can be rewritten as follows:

$$cov_{ye}\left(t,\;\bar{A}\right)=\frac{\partial }{\partial \bar{A}}\bar{y}\left(t,\;\bar{A}\right)\cdot \sigma_{A}^{2}$$ (9)

$$var_{y}\left(t,\;\bar{A}\right)={\left[\frac{\partial }{\partial \bar{A}}\bar{y}\left(t,\;\bar{A}\right)\right]}^{2}\cdot \sigma_{A}^{2}$$ (10)

Note that by definition $\overset{-}{y}(\overset{-}{D},\;\overset{-}{A})\;=\;\overset{-}{A}$, and $\frac{\partial }{\partial \overset{-}{A}}\overset{-}{y}(\overset{-}{D},\;\overset{-}{A})\;=\;1$. Therefore, planning noise is characterized by a correlation trajectory Eq (2) which rises to 1 immediately at saccade onset. Of course, this is predicted only in the hypothetical absence of any other noise sources. As noted above, adding motor noise (or external noise) will cause the correlation trajectory to raise more slowly. Using the two eqs 9 and 10, the planning noise associated with a given mean ($\overset{-}{A}$) and variance ($\sigma_{A}^{2}$) of the saccade amplitude was computed for each individual by estimating the partial derivative $\frac{\partial }{\partial \overset{-}{A}\;}\overset{-}{y\;}(t,\;\overset{-}{A})$. This derivative was approximated by the empirical difference quotient

$$\frac{\partial }{\partial \bar{A}}\bar{y}\left(t,\;\bar{A}\right)\approx \frac{\bar{y}\left(t,\;\bar{A}+\Delta /2\right)-\bar{y}\left(t,\;\bar{A}-\Delta /2\right)}{\Delta },$$ (11)

where the two main–sequence trajectories $\overset{-}{y}(t,\;\overset{-}{A}\pm \Delta /2)$ were evaluated by averaging across all available saccades with amplitudes within the ranges $\left[\overset{-}{A}-\Delta ,\;\overset{-}{A}\right]$ and $\left[\overset{-}{A},\;\overset{-}{A}+\Delta \right]$. The bin-width was chosen as Δ = 5 deg. In accordance with our definitions of the dependent variables in the section “Inter-trial variability of saccade trajectories”, the time (*t*) in all the above equations denotes the normalized time axis $t\in [0,\;\overset{-}{D}]$. Thus, to estimate the variance and covariance trajectories induced by planning noise, using Eqs 9 and 10 corresponds to the definitions of the experimentally obtained estimates of ${var}_{y}(t,\;\overset{-}{A})$ and ${cov}_{ye}(t,\;\overset{-}{A})$.

Obviously, the variance of the planned saccade amplitude in our empirical data samples selected for each class of motor errors is not only due to variability in the planning process alone, but also to the variability of the motor error within the selected saccade class. However, the covariance and variance trajectories given by Eqs 9/10 depend only on the variance ($\sigma_{A}^{2}$) of the amplitude due to planning noise, no matter whether the source of this variance is internal or external.

The standard deviation of the mean amplitude, due to planning noise, is likely to increase linearly with increasing target eccentricity because the magnification factor of spatiotopic neuronal maps increase linearly with horizontal eccentricity in sensory maps such as the primary visual cortex [34], as well as in motor maps such as the SC [15]. Therefore, we assumed (see S1 Text) that the variance of the saccade amplitude scales with the square of the mean saccade amplitude:

$$\sigma_{A}^{2}=k_{A}^{2}\cdot {\bar{A}}^{2}$$ (12)

Because the mean saccade amplitude $\overset{-}{A}$, the main sequence trajectory $\overset{-}{y}(t,\;A)$ as well as their derivative $\frac{\partial }{\partial \overset{-}{A}\;}\overset{-}{y\;}(t,\;\overset{-}{A})$ were estimated from the measured data (separately for each monkey and for each saccade condition), the prediction of the effects of planning noise on the variance and covariance trajectories by Eqs (9) and (10) is determined by *k*_*A*_, the coefficient of variation of the planned saccade amplitude. This parameter, which we will call *planning-noise coefficient* for the sake of brevity, is the only free parameter of this model of planning noise.

#### Signal-dependent noise in the oculomotor neurons

The second source of inter-trial variability was adopted from the model proposed by Harris and Wolpert [2, 3]. As in this study, the control signal (*C*_3_(*t*), Fig 10) driving the oculomotor neurons was assumed to be contaminated by white Gaussian noise (*r*_*ON*_(*t*)) resulting from the multiplication of a standard white Gaussian noise (power density: 1/Hz) with the signal-dependent factor *k*_*ON*_∙*C*_3_(*t*). This noise predicts a covariance trajectory which equals

$$cov_{ye}\left(t\right)=\int_{\tau =0}^{t}Q_{ON}\left(\tau \right)\cdot p\left(t-\tau \right)\cdot p\left(D-\tau \right)\;d\tau ,$$ (13)

where *D* denotes the saccade duration, *p*(*t*) the impulse response of the linear plant, and

$$Q_{ON}\left(t\right)=k_{ON}^{2}\cdot \left({\bar{C}}_{3}^{2}\left(t\right)+var_{C_{3}}\left(t\right)\right)$$ (14)

denotes the power density of the signal-dependent noise *r*_*ON*_. The variance trajectory equals

$$var_{y}\left(t\right)=\int_{\tau =0}^{t}Q_{ON}\left(\tau \right)\cdot p^{2}\left(t-\tau \right)\;d\tau .$$ (15)

In the special case of the model considered by Harris and Wolpert [3], the control signal *C*_3_(*t*) was assumed to be a deterministic signal and thus, it was assumed that $var_{C_{3}}\left(t\right)=0$. Therefore, the power density of the signal-dependent noise *r*_*ON*_ could be expressed by $Q_{ON}\left(t\right)=k_{ON}^{2}\cdot {\bar{C}}_{3}^{2}\left(t\right)$ (see Harris and Wolpert [3], Eq 7). However, since we extended this noise model by an additional noise source due to signal-dependent noise added to the activity of the PBNs, *C*_3_(*t*) had to be considered as a random signal, and therefore it was necessary to use the more complicated Eq 14. The supporting information (S2 Text) provides a derivation of Eqs 13–15. In analogy to Eq 15, the variance of the control signal *C*_3_(*t*) was computed by the convolution

$$var_{C_{3}}\left(t\right)=\int_{\tau =0}^{t}Q_{PBN}\left(\tau \right)\cdot n^{2}\left(t-\tau \right)\;d\tau ,$$ (16)

where *n*(*t*) denotes the impulse response of the linear system transforming the signal-dependent noise *r*_*PBN*_ into the random component of the control signal *C*_3_(*t*) (note that the transfer function corresponding to *n*(*t*) is the product of the transfer functions of the closed loop control circuit around the PBN and the pulse-step mechanism). *Q*_*PBN*_(*t*) denotes the power density of *r*_*PBN*_.

The eqs 13–16 show that the noise in the saccade trajectory caused by signal-dependent noise in the ONs is modulated by $var_{C_{3}}\left(t\right)$, which depends on noise entered in the PBN. However, the effect of the noise in structures upstream from the ON on the signal-dependent noise in the ON is limited to a modulation of the power density *Q*_*ON*_(*t*) Eq (14) which is not a random, but a deterministic signal. For given power densities *Q*_*ON*_(*t*) and *Q*_*PBN*_(*t*), the model is linear in *r*_*ON*_ and *r*_*PBN*_, and both of these signal-dependent noises are assumed to be independent of each other. Therefore, both variance and covariance trajectories of the saccadic eye position can be computed by linear superposition of the variance and covariance trajectories induced by the two sources of signal-dependent noise. Eqs 13–16 model the noise components due to signal-dependent noise in the ON. The three free parameters of this model are *k*_*ON*_, the feedback gain *g*, and *k*_*PBN*_, whereby *g* is needed to compute the impulse response *n*(*t*), and both *g* and *k*_*PBN*_ to compute *Q*_*PBN*_(*t*) which is described in the following section.

#### Signal-dependent noise in the brainstem saccade generator

Eqs 13–15 can be applied analogously for the noise component of the eye position due to signal-dependent noise *r*_*PBN*_ added to the PBN activity (as shown in the left part of Fig 10).

$$cov_{ye}\left(t\right)=\int_{\tau =0}^{t}Q_{PBN}\left(\tau \right)\cdot q\left(t-\tau \right)\cdot q\left(\text{D}-\tau \right)\;d\tau$$ (17)

$$Q_{PBN}\left(t\right)=k_{PBN}^{2}\cdot \left({\bar{C}}_{2}^{2}\left(t\right)+var_{C_{2}}\left(t\right)\right)$$ (18)

$$var_{y}\left(t\right)=\int_{\tau =0}^{t}Q_{PBN}\left(\tau \right)\cdot q^{2}\left(t-\tau \right)\;d\tau ,$$ (19)

where *q*(*t*) denotes the impulse response of the linear system transforming the signal-dependent noise *r*_*PBN*_ into the corresponding noise component of eye position, and equals the convolution

q(t)=n(t)*p(t), (20)

where *n*(*t*)and *p*(*t*) are defined as in the last section. The situation is complicated by the fact that Eq 18 cannot directly be evaluated because, due to the feedback, ${var}_{C_{2}}(t)$, i.e. the variance of the control signal *C*_2_(*t*), depends in turn on the power density *Q*_*PBN*_(*t*).

$$var_{C_{2}}\left(t\right)=\int_{\tau =0}^{t}Q_{PBN}\left(\tau \right)\cdot m^{2}\left(t-\tau \right)\;d\tau ,$$ (21)

where *m*(*t*) denotes the impulse response of the closed loop system transferring the signal-dependent noise *r*_*PBN*_ to the noise component of *C*_2_. Inserting Eq 21 in Eq 18 shows that *Q*_*PBN*_(*t*) is the solution of a linear Volterra integral equation of second type [50]:

$$Q_{PBN}\left(t\right)=k_{PBN}^{2}\cdot {\bar{C}}_{2}^{2}\left(t\right)+k_{PBN}^{2}\cdot \int_{\tau =0}^{t}Q_{PBN}\left(\tau \right)\cdot m^{2}\left(t-\tau \right)\;d\tau$$ (22)

For given *k*_*PBN*_, ${\overset{-}{C}}_{2}(t)$, and *m*(*t*), a time discrete approximation of *Q*_*PBN*_(*t*) was computed by recursion of

$$Q_{PBN}\left(n\cdot \Delta t\right)=\frac{k_{PBN}{}^{2}\cdot {\bar{C}}_{2}^{2}\left(t\right)+k_{PBN}^{2}\cdot \sum_{i=0}^{n-1}Q_{PBN}\left(i\cdot \Delta t\right)\cdot m^{2}\left(\left(n-i\right)\cdot \Delta t\right)\cdot \Delta t}{1-k_{PBN}^{2}\cdot m^{2}\left(0\right)\cdot \Delta t},$$ (23)

starting with $Q_{PBN}(0)\;=\;k_{PBN}^{2}∙{\overset{-}{C}}_{2}^{2}(0)$.

The two free parameters of this noise component are *k*_*PBN*_ and the feedback gain *g* (needed to compute *n*(*t*) and *m*(*t*)).

#### Model fitting and model comparison

For a given set of the free model parameters [*k*_*A*_, *k*_*ON*_, *k*_*PBN*_, *g*] and the mean saccade trajectory $\overset{-}{y}(t,\;\overset{-}{A})$, estimated separately for each monkey and for each condition, the corresponding mean control signals ${\overset{-}{C}}_{2}(t)$, and ${\overset{-}{C}}_{3}(t)$, the variance trajectories (*var*_*y*_(*t*)), and the covariance trajectories (*cov*_*ye*_(*t*)) were computed for each of the three different noise sources described above. The sum of these three variance/covariance trajectories was subtracted from the corresponding measured variance/covariance trajectory of the real eye movement data, and two mean squared error parts were computed from these differences (one for the variance trajectory, and the second for the covariance trajectory). Fitting the model was achieved by systematic variation in [*k*_*A*_, *k*_*ON*_, *k*_*PBN*_, *g*] in order to minimize the mean square error (MSE [deg^4^]) of the model, defined as the mean of the two mean squared error parts. The numerical minimization was performed using the MATLAB^®^-function *fmincon*. Overall fit quality was quantified by the coefficient of determination $R^{2}\;=\;1-\frac{MSE}{{VAR}_{tot}}$, where *VAR*_*tot*_ [deg^4^] denotes the mean of the total variances of the two fitted trajectories.

To test whether our assumption of signal-dependent noise of a PBN within an internal feedback loop constitutes a significant improvement compared to the motor noise model proposed by Harris and Wolpert [3], in addition to the full model with the 4 parameters [*k*_*A*_, *k*_*ON*_, *k*_*PBN*_, *g*], two reduced versions of the model were fitted. In the first reduced version (without PBN noise), the signal-dependent noise of the PBN was set to zero by fixing *k*_*PBN*_ = *Q*_*PBN*_ = 0. As a consequence of this constraint both variance and covariance trajectories become independent of the feedback gain (*g*) and only the two parameters [*k*_*A*_, *k*_*ON*_] remain to be fitted in that case. The second reduced version (without ON noise) assumed the absence of any signal-dependent noise in the ON by setting *k*_*ON*_ = *Q*_*ON*_ = 0 and three parameters [*k*_*A*_, *k*_*PBN*_, *g*] remained to be fitted in this model. Model comparison was performed using the Akaike information criterion [51] computed as

$$AIC=N_{obs}\cdot \text{ln}\left(MSE\right)+2\cdot N_{par}$$ (24)

where *N*_*obs*_ denotes an estimate of the number of independent observations underlying the measured data, and *N*_*par*_ the number of fitted parameters. The difference of *AIC* between any model and the best model (i.e., the model with the smallest *AIC*)

$$\Delta AIC_{\text{i}}=AIC_{i}-AIC_{min}$$ (25)

was used as a criterion to evaluate the empirical support of model i according to the classification featured by Burnham and Anderson [52] (page. 70): Small differences (Δ*AIC*_i_ < 2) were considered to provide “substantial”, intermediate differences (4 < Δ*AIC*_i_ < 7) “considerably less”, and large differences (Δ*AIC*_i_ > 10) “essentially no empirical support of model i".

The relative contribution of *N*_*par*_ to the criterion Eq 24 decreases with increasing *N*_*obs*_. Therefore, overestimating *N*_*obs*_ has the consequence of assigning empirical support to overfitted models. In the present case, where the underlying measured data are the two time courses of the variance and covariance trajectories, the estimate of *N*_*obs*_ may be considered problematic. For this reason, we set *N*_*obs*_ = 6, thereby adopting a very conservative strategy which assumed only 3 independent observations per trajectory (i.e., the minimum for a time course being distinguished from a straight line).

Statistical comparison of the MSE between models was performed by submitting the log-transformed MSE to a repeated measures ANOVA with the factor *Model* repeated within each monkey. The log-transformation was applied to meet the normality assumption of this parametric test of the inherently non-normally distributed MSE. The normality of the log-transformed MSE was verified using the Lilliefors test. The sphericity assumption of the repeated measures ANOVA was verified with the Mauchly-test. Scheffé’s post hoc test was used to evaluate pairwise differences of the log(MSE) between factor levels. Effects with α-errors smaller than 0.05 are considered significant.

## Supporting Information

S1 Text Signal dependency of planning noise. (PDF)

S2 Text Transfer of signal-dependent noise through linear systems. (PDF)

S3 Text The characteristic difference between motor noise entering within or downstream from a premotor feedback loop. (PDF)
