# Supplementary material for: Modeling Inter-trial Variability of Saccade Trajectories: Effects of Lesions of the Oculomotor Part of the Fastigial Nucleus
Source: PLoS Comput Biol. 2016 Jun 28;12(6):e1004866. doi: 10.1371/journal.pcbi.1004866 (PMC4924843; doi:10.1371/journal.pcbi.1004866)
Supplement: S1 Text — (PDF) [file pcbi.1004866.s001.pdf]

S1 Text: Signal dependency of planning noise.

This section shows that Eq. 12 reflects the assumption that position signals in sensory areas or in higher motor areas are coded in homogeneous spatiotopic maps. For example, the center of neuronal activity in the spatiotopic map in the SC encodes a motor command in retinal coordinates [53], where, for pure horizontal movements, the motor plan (MP, expressed in degrees of desired movement angle) depends exponentially on the map position (u [mm]) of the activity on the SC [15]:

$$MP = A \cdot \exp\left(\frac{u}{B_u}\right) - A \qquad , \qquad \text{(A1)}$$

with some scaling factors A and B_u. For large motor commands, i.e. $exp\left(\frac{u}{B_u}\right) \gg 1$, the map scaling function may be approximated by

$$MP \approx A \cdot \exp\left(\frac{u}{B_u}\right) \qquad . \qquad \text{(A2)}$$

The corresponding magnification factor of the map (MF_{map}), computed as the derivative of the exponential scaling function (A2) with respect to the map position, scales linearly with the mean motor plan $\overline{MP}$:

$$MF_{map} = \frac{d}{d\,u}\left[\mathrm{A} \cdot \exp\left(\frac{u}{B_u}\right)\right]\Bigg|_{u=\bar{u}} = \frac{A}{B_u} \cdot \exp\left(\frac{\bar{u}}{B_u}\right) \approx \frac{1}{B_u} \cdot \overline{MP} \qquad . \qquad \text{(A3)}$$

Assuming that the planning noise originates from a constant inter-trial standard deviation on the map (σ_u), independent of the position on the map, the variance (σ_{MP}^2) of the motor plan (MP) scales with the square of its mean ($\overline{MP}$).

$$\sigma_{MP}^2 = \sigma_u^2 \cdot MF_{map}^2 = \left(\frac{\sigma_u}{B_u}\right)^2 \cdot \overline{MP}^2 \qquad . \qquad \text{(A4)}$$

Because the saccade amplitude is assumed to be proportional to the motor plan ($A = G \cdot MP$), it follows from Eq. A4 that

$$\sigma_A^2 = G^2 \cdot \sigma_{MP}^2 = G^2 \cdot \left(\frac{\sigma_u}{B_u}\right)^2 \cdot \overline{MP}^2 = \left(\frac{\sigma_u}{B_u}\right)^2 \cdot \bar{A}^2 \qquad . \tag{A5}$$

Eq. 12 follows from Eq. A5 by identifying the noise coefficient k_A with

$$k_A = \frac{\sigma_u}{B_u} \qquad . \tag{A6}$$

Thus, the noise coefficient k_A is proportional to the inter-trial standard deviation of the activated map position.
