# Supplementary material for: Modeling Inter-trial Variability of Saccade Trajectories: Effects of Lesions of the Oculomotor Part of the Fastigial Nucleus
Source: PLoS Comput Biol. 2016 Jun 28;12(6):e1004866. doi: 10.1371/journal.pcbi.1004866 (PMC4924843; doi:10.1371/journal.pcbi.1004866)
Supplement: S2 Text — (PDF) [file pcbi.1004866.s002.pdf]

S2 Text: Transfer of signal-dependent noise through linear systems.

This section provides a short derivation for the relations between the impulse response $p(t)$ of a linear system, the power density $Q(t)$ of a signal-dependent noise added to its input, and the variance and covariance trajectories of its output as summarized in Equations 13-15.

Continuous Gaussian white noise $w(t)$ with zero mean and unity power density is characterized by

$$E\{w(t)\} = 0 \quad , \tag{A7}$$

and an autocovariance function that equals a Dirac delta function:

$$ACV_{ww}(t_1, t_2) = E\{w(t_1) \cdot w(t_2)\} = \delta(t_1 - t_2) \quad . \tag{A8}$$

Signal-dependent noise generated by multiplication of such a continuous Gaussian white noise with a control signal u

$$r_u(t) = k \cdot u(t) \cdot w(t) \tag{A9}$$

has the autocovariance function

$$\begin{aligned} ACV_{rr}(t_1, t_2) &= E\{r_u(t_1) \cdot r_u(t_2)\} \\ &= k^2 \cdot E\{u(t_1) \cdot u(t_2) \cdot w(t_1) \cdot w(t_2)\} \quad . \\ &= k^2 \cdot E\{u(t_1) \cdot u(t_2)\} \cdot \delta(t_1 - t_2) \end{aligned} \tag{A10}$$

This signal-dependent noise is transferred through a linear system with the impulse response $p(t)$ by the convolution

$$y(t) = \int_{\tau=0}^{t} r_u(\tau) \cdot p(t - \tau) d\tau \quad . \tag{A11}$$

The autocovariance function of this response is

$$ACV_{yy}(t_1,t_2) = E\{y(t_1)\cdot y(t_2)\}$$

$$= \iint_{\tau_1,\tau_2=0}^{t_1,t_2} E\{r_u(\tau_1)\cdot r_u(\tau_2)\}\cdot p(t_1-\tau_1)\cdot p(t_2-\tau_2)\, d\tau_2\, d\tau_1 \quad . \qquad \text{(A12)}$$

$$= \iint_{\tau_1,\tau_2=0}^{t_1,t_2} ACV_{rr}(\tau_1,\tau_2)\cdot p(t_1-\tau_1)\cdot p(t_2-\tau_2)\, d\tau_2\, d\tau_1$$

Using the definition of the noise autocovariance (Eq. A10), we obtain

$$ACV_{yy}(t_1,t_2) = \int_{\tau_1=0}^{t_1}\left[\int_{\tau_2=0}^{t_2} k^2\cdot E\{u(\tau_1)\cdot u(\tau_2)\}\cdot \delta(\tau_1-\tau_2) \cdot p(t_2-\tau_2)\; d\tau_2\right]\cdot p(t_1-\tau_1)\, d\tau_1 \quad . \qquad \text{(A13)}$$

The integral in the square bracket is easily solved since its integrand forms a product with the dirac delta function as one factor:

$$ACV_{yy}(t_1,t_2) = \int_{\tau=0}^{t_1} Q(\tau)\cdot p(t_1-\tau)\cdot p(t_2-\tau)\, d\tau \quad , \qquad \text{(A14)}$$

where

$$Q(t) := k^2\cdot E\{u^2(t)\} \qquad \text{(A15)}$$

defines the time variant power density of the signal $r_u(t)$. We obtain Eq. 13 from Eq. A14 through

$$ACV_{yy}(t,t_e) = E\{y(t)\cdot y(t_e)\} = cov_{ye}(t) \quad , \qquad \text{(A16)}$$

Eq. 14 from Eq. A15 by using $E\{u^2(t)\} = \bar{u}^2(t) + var_u(t)$ and substituting the control signal $C_3(t)$ for $u(t)$. Eq. 15 is obtained from Eq. A14 by considering that

$$ACV_{yy}(t,t) = E\{y(t)\cdot y(t)\} = var_y(t) \quad . \qquad \text{(A17)}$$
