# Supplementary material for: Modeling Inter-trial Variability of Saccade Trajectories: Effects of Lesions of the Oculomotor Part of the Fastigial Nucleus
Source: PLoS Comput Biol. 2016 Jun 28;12(6):e1004866. doi: 10.1371/journal.pcbi.1004866 (PMC4924843; doi:10.1371/journal.pcbi.1004866)
Supplement: S3 Text — (PDF) [file pcbi.1004866.s003.pdf]

S3 Text: The characteristic difference between motor noise entering within or downstream from a premotor feedback loop.

Our simulations showed that ON-noise caused a monotonically increasing variance of eye position during the saccade whereas PBN-noise induced transient, bell-shaped pattern of the variance. We show in the following that this characteristic difference between motor noise entering within or downstream from a premotor feedback loop does not depend on the fine-tuning of plant parameters. This characteristic difference holds for a large class of models as long as they fulfill few conditions.

Signal dependent noise in the ON leads to monotonically increasing variance of eye position during the saccade. The only condition to be fulfilled to ensure this feature is that the plants must be a low pass with a dominant time constant which is longer than the duration of the saccade. This can be seen from Eq. 15:

$$var_y(t) = \int_{\tau=0}^{t} Q_{ON}(\tau) \cdot p^2(t-\tau)\ d\tau$$

Differentiating leads to

$$\frac{d}{dt} var_y(t) = \int_{\tau=0}^{t} 2 \cdot Q_{ON}(\tau) \cdot p(t-\tau) \cdot \dot{p}(t-\tau)\ d\tau \qquad . \qquad \text{(A18)}$$

The impulse response $p(t)$, as well as its derivative $\dot{p}(t)$ are positive during the saccade ($t < D$) because of the required low-pass characteristic of the plant. $Q_{ON}(t)$ is positive by definition. Thus, the above derivative is positive. This shows that the resulting variance of eye position during the saccade increases monotonically. Interestingly, this conclusion does not depend either on the time course of the activity of the ON, nor on the fine tuning of the plant parameters.

Signal dependent noise entering inside of a premotor feedback loop causes a transient pattern of variance of eye position with a duration shorter than the saccade duration *D*. For the system shown in Fig 10 without delay in the feedback loop ($\Delta t = 0$), we can define four conditions that are sufficient for this conclusion:

1) The linear dynamics (defined by the impulse response $q(t)$) transforming the PBN-noise $r_{PBN}(t)$ to eye position (see Eq. 20) is a low pass with a dominant time constant which is shorter than *D*.

2) The coefficient of variation of the PBN-noise must not exceed an upper limit:

$$k_{PBN} < \sqrt{D} \quad . \qquad \text{(A19)}$$

3) The gain *g* must be inside the range,

$$g \in \frac{1}{k_{PBN}^2} \pm \frac{\sqrt{1/k_{PBN}^2 - 1/D}}{k} \quad . \qquad \text{(A20)}$$

4) The duration of the mean burst activity $\overline{C}_2(t)$ and its derivative $\dot{\overline{C}}_2(t)$ must be smaller *D*.

To demonstrate that these four conditions are sufficient for our conclusion we consider Eq. 19:

$$var_y(t) = \int_{\tau=0}^{t} Q_{PBN}(\tau) \cdot q^2(t-\tau)\ d\tau$$

One can see that the variance $var_y(t)$ is transient with a duration smaller than *D* if the duration of both the square of the impulse response $q(t)$ and the duration of the power density of the PBN-noise $Q_{PBN}(t)$ are smaller than *D*. Condition 1) ensures that $q(t)$ is short enough. Conditions 2), 3) and 4) ensure that the duration $Q_{PBN}(t)$ is smaller than *D*. This is shown by

transforming Eq. 22 from a Volterra integral equation into an ordinary differential equation for $Q_{PBN}(t)$ (which is achieved by differentiating Eq. 22 with respect to *t*):

$$\dot{Q}_{PBN}(t) = -\frac{1}{\tau_Q} \cdot Q_{PBN}(t) + I_Q(t) \quad . \tag{A21a}$$

with

$$\frac{1}{\tau_Q} = 2 \cdot g - g^2 \cdot k_{PBN}^2 \tag{A21b}$$

and

$$I_Q(t) = 2 \cdot k_{PBN}^2 \cdot \overline{C}_2(t) \cdot \left(\dot{\overline{C}}_2(t) + g \cdot \overline{C}_2(t)\right) \tag{A21c}$$

A detailed derivation of Eq. A21 is provided below (see section “Transformation of the Volterra integral equation”). The solution of the differential equation A21 is

$$Q_{PBN}(t) = Q_{PBN}(t) \cdot \exp\left(-\frac{t-\tau}{\tau_Q}\right) + \int_{\tau=0}^{t} I_Q(\tau) \cdot \exp\left(-\frac{t-\tau}{\tau_Q}\right) d\tau \quad . \tag{A22}$$

This shows that the duration of $Q_{PBN}(t)$ is shorter than the saccade duration *D* if the duration of both $\exp\left(-\frac{t-\tau}{\tau_Q}\right)$ and $I_Q(t)$ are clearly shorter than *D*. Condition 2) and 3) ensure that τ_Q and thereby also the duration of $\exp\left(-\frac{t-\tau}{\tau_Q}\right)$ is shorter than *D*. Condition 4) ensures that the duration of $I_Q(t)$ is shorter than *D*. Thus, also our second main conclusion does not depend either on the fine tuning of the plant or on the exact shape of $\overline{C}_2(t)$.

Note: In this section the term "The duration of function f(t) is shorter than D." means that the majority of the area under the function appears for $t < t_{max}$*:*

$$\frac{\int_{t=0}^{t_{max}} |f(\tau)|\, d\tau}{\int_{t=0}^{\infty} |f(\tau)|\, d\tau} > 1 - \alpha \qquad \text{with} \qquad t_{max} < D, \qquad \alpha \ll 1 \quad .$$

It is not necessary to assume that

$$f(t > t_{max}) = 0 \quad \text{with} \quad t_{max} < D \quad .$$

Thus, the example of $\overline{C}_2(t)$ *as shown in Fig 11D fulfills Condition 3.*

Transformation of the Volterra integral equation

The power density of the PBN activity as a function of time is defined by the integral equation Eq. 22:

$$Q_{PBN}(t) = k_{PBN}^2 \cdot \bar{C}_2^2(t) + k_{PBN}^2 \cdot \int_{\tau=0}^{t} Q_{PBN}(\tau) \cdot m^2(t-\tau)\ d\tau \quad ,$$

with

$$Q_{PBN}(0) = k_{PBN}^2 \cdot \bar{C}_2^2(0) \quad , \quad \text{(A23)}$$

where $m(t)$ denotes the impulse response of the dynamics transforming the noise $r_{PBN}(t)$ into the control signal $C_2(t)$ (see Fig 10).

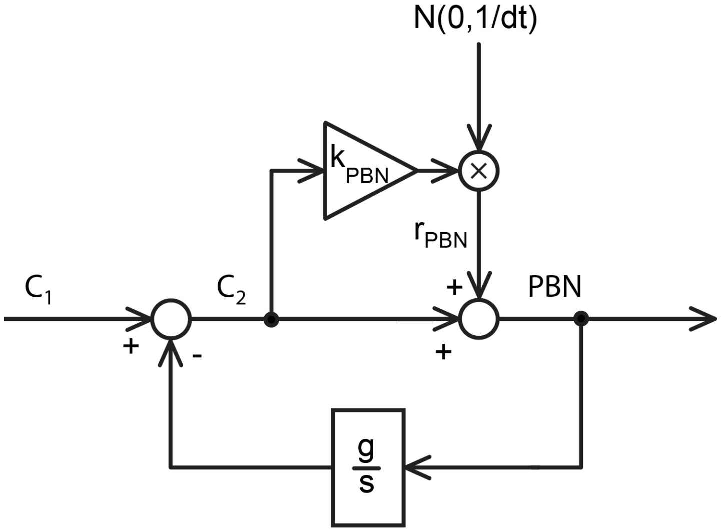

Fig A1: Signal dependent noise entering inside of a feedback loop.: deterministic input to the feedback loop. r_PBN: signal dependent noise. PBN: Burst signal. C_2: random control signal driving the burster.

In the simplified case that the feedback loop does not contain a delay (see Fig A1), the integral equation (Eq. 22) can easily be transformed in to an ordinary differential equation

because $m(t)$ is in that case the impulse response of a simple low pass with the Laplace transfer function

$$M(s) = \frac{-\frac{g}{s}}{1+\frac{g}{s}} = -\frac{1}{\frac{s}{g}+1} \qquad . \qquad \text{(A24)}$$

The impulse response of this low pass is

$$m(t) = -g \cdot exp\,(-g \cdot t) \qquad . \qquad \text{(A25)}$$

Inserting Eq. A25 in the Volterra integral equation (Eq. 22) and differentiating leads to

$$\begin{aligned} \dot{Q}_{PBN}(t) &= 2 \cdot k_{PBN}^2 \cdot \overline{C}_2(t) \cdot \dot{\overline{C}}_2(t) \\ &\quad + \frac{d}{dt}\left[k_{PBN}^2 \cdot g^2 \int_{\tau=0}^{t} Q_{PBN}(\tau) \cdot exp\big(-2 \cdot g \cdot (t-\tau)\big)\, d\tau\right] \\ &= 2 \cdot k_{PBN}^2 \cdot \overline{C}_2(t) \cdot \dot{\overline{C}}_2(t) + k_{PBN}^2 \cdot g^2 \cdot Q_{PBN}(t) \\ &\quad - 2 \cdot k_{PBN}^2 \cdot g^3 \cdot \int_{\tau=0}^{t} Q_{PBN}(\tau) \cdot exp\big(-2 \cdot g \cdot (t-\tau)\big)\, d\tau \end{aligned} \qquad . \qquad \text{(A26)}$$

Using Eq. 22, the last term of A26 can be identified as

$$\begin{aligned} &2 \cdot k_{PBN}^2 \cdot g^3 \cdot \int_{\tau=0}^{t} Q_{PBN}(\tau) \cdot exp\big(-2 \cdot g \cdot (t-\tau)\big)\, d\tau \\ &\quad = 2 \cdot g \cdot Q_{PBN}(t) - 2 \cdot g \cdot k_{PBN}^2 \cdot \overline{C}_2^2(t) \end{aligned} \qquad . \qquad \text{(A27)}$$

Inserting Eq. A27 in A26 yields

$$\begin{aligned} \dot{Q}_{PBN}(t) &= 2 \cdot k_{PBN}^2 \cdot \overline{C}_2(t) \cdot \dot{\overline{C}}_2(t) + k_{PBN}^2 \cdot g^2 \cdot Q_{PBN}(t) \\ &\quad - 2 \cdot g \cdot Q_{PBN}(t) + 2 \cdot g \cdot k_{PBN}^2 \cdot \overline{C}_2^2(t) \end{aligned} \qquad . \qquad \text{(A28)}$$

By appropriate grouping

$$\dot{Q}_{PBN}(t) = (k_{PBN}^2 \cdot g^2 - 2 \cdot g) \cdot Q_{PBN}(t) + 2 \cdot k_{PBN}^2 \cdot \overline{C}_2(t) \cdot \left(\dot{\overline{C}}_2(t) + g \cdot \overline{C}_2(t)\right) \qquad \text{(A29)}$$

it turns out that Eq. A29 is an ordinary first order differential equation. Comparing the coefficients of Eq. A29 with that of Eq. A21 shows that both are identical.
